# Inferring laminar origins of MEG signals with optically pumped magnetometers (OPMs): A simulation study

**DOI:** 10.1162/imag_a_00410

**Published:** 2025-01-02

**Authors:** Saskia Helbling

**Affiliations:** Ernst Strüngmann Institute for Neuroscience in Cooperation with Max Planck Society, Frankfurt am Main, Germany

**Keywords:** magnetoencephalography, optically pumped magnetometers, laminar inference, source reconstruction, simulation study

## Abstract

We explore the potential of optically pumped magnetometers (OPMs) to non-invasively infer the laminar origins of neural activity. OPM sensors can be positioned closer to the scalp than conventional cryogenic magnetoencephalography (MEG) sensors, opening an avenue to higher spatial resolution when combined with high-precision source space modelling. By simulating the forward model projection of single dipole sources at deep and superficial cortical surfaces onto OPM sensor arrays with varying sensor densities and measurement axes, and employing sparse source reconstruction approaches, we find that laminar inference with OPM arrays is possible at relatively low sensor counts under moderate-to-high signal-to-noise ratios (SNR). We observe improvements in laminar inference with increasing spatial sampling densities and measurement axes and demonstrate the advantage of placing the sensors closer to the scalp for inferring the laminar origins of cortical sources. However, challenges remain, such as biases towards both the superficial and deep surfaces at very low SNRs and a notable bias towards the deep surface when combining empirical Bayesian beamformer (EBB) source reconstruction with a whole-brain analysis. Adequate SNR through appropriate trial numbers and shielding, as well as precise co-registration, is crucial for reliable laminar inference with OPMs.

## 1 Introduction

Magnetoencephalography (MEG) is a non-invasive technique that measures the tiny magnetic fields generated by the synchronous current flow through neuronal populations in the brain (Cohen, 1968;Hämäläinen & Ilmoniemi, 1994). Conventional MEG operates with superconducting SQUID magnetometers that must be immersed in liquid helium for cooling and are therefore placed within a fixed dewar for thermal insulation. This results in a substantial (several centimetres) gap between the sensors and the scalp and, as the strength of the magnetic field decreases with distance from the source, weaker magnetic fields at the sensor locations.

Optically pumped magnetometers (OPMs) are highly sensitive magnetometers that operate without the need for cryogenic cooling. The sensors are small and lightweight and can therefore be flexibly arranged on, and placed close to, the scalp. OPMs contain helium gas (Fourcault et al., 2021) or a vapour of alkali atoms (Allred et al., 2002;Colombo et al., 2016;Kowalczyk et al., 2021) whose atomic spins are aligned through optical pumping. Fluctuations in the local magnetic field affect the transmission of laser light through this spin-polarised gas or vapour and can be measured as changes in the amount of light at a photodetector site (Brookes et al., 2022;Nugent et al., 2022). This new technology has sparked immense interest in the MEG community, and the first whole-head systems are now commercially available.

By placing the sensors directly on the scalp, the distance between the sensors and the cortical sources is decreased. Simulations show that bringing the sensors closer to the participant’s brain substantially improves the sensitivity to cortical sources and enables the sampling of higher spatial frequencies, that is, this permits measurement of more focal field patterns, resulting in improved spatial resolution and better source separability than conventional MEG (Boto et al., 2016;Iivanainen et al., 2017;Nugent et al., 2022). Experimental studies show that OPM-MEG systems exhibit comparable or even larger signal amplitudes and improved source reconstruction accuracy than conventional SQUID-MEG systems, even when they had fewer sensors with higher noise floors (Borna et al., 2020;Hill et al., 2020;Iivanainen et al., 2019;Sander et al., 2012).

A significant challenge in reconstructing the brain sources from MEG measurements taken on the scalp is to distinguish between sources originating from different cortical layers. Non-invasive laminar electrophysiology in humans would be of high relevance for both basic and clinical research, in particular in neurodegenerative diseases such as Huntington’s disease (Rüb et al., 2016) and multiple sclerosis (Derakhshan et al., 2014;Tardif et al., 2012) where different layers are affected across disease stages, and in investigating cortical microcircuits and inter-laminar neural communication (Scheeringa et al., 2016). However, with a cortical thickness of 2–5 mm (von Economo & Koskinas, 1925;Wagstyl et al., 2020), the typical spatial resolution achieved by conventional MEG (Barnes et al., 2004;Hauk et al., 2022) is not sufficient for laminar inference.

It is possible to distinguish between deep and superficial sources by using forward models with high-precision cortical surfaces as source space (Bonaiuto, Rossiter, et al., 2018;Meyer et al., 2017;Troebinger, López, Lutti, Bestmann, & Barnes, 2014;Troebinger, López, Lutti, Bradbury, et al., 2014). Here, the main idea is to exploit the small variations in the so-called lead fields between deep and superficial sources to infer the more likely origin of the source activity, by comparing the model evidence of models that place the source activity in either the deep or the superficial layers. Such laminar forward models have been used to accurately infer the origin of cortical dipole sources in simulations, applied experimentally to a visuo-motor paradigm (Bonaiuto, Meyer, et al., 2018), and have been successfully used to derive laminar contributions to beta bursts in a temporally resolved manner (Bonaiuto et al., 2021).

On-scalp OPM-MEG has been postulated to have the potential to further improve the discriminability of laminar sources (Bonaiuto, Rossiter, et al., 2018;Bonaiuto et al., 2021;Ihle et al., 2020) as laminar inference was more successful for sources close to the sensors (Bonaiuto, Rossiter, et al., 2018). To investigate the conditions under which OPMs can improve the performance of laminar inference, we simulate cortical sources at deep and superficial layers and infer their laminar origin using forward models with deep and superficial surfaces as source space. We explore the impact of two main features of OPM sensor arrays: the number of sensors and the number of measurement axes. While a higher sensor density yields a higher spatial resolution in source reconstruction (Boto et al., 2016;Clancy et al., 2021;Iivanainen et al., 2017;Nugent et al., 2022;Vrba et al., 2004), it raises questions of feasibility due to the potential for crosstalk and heating issues. OPM sensors with multiple measurement axes provide enhanced information capacity (Iivanainen et al., 2017) and have been shown to reduce external interference and motion artefacts substantially (Boto et al., 2022;Brookes et al., 2021). Additionally, the different sensitivity profiles of triaxial sensors allow for better and more uniform coverage, particularly for children (Boto et al., 2022). We also investigate the effects of varying signal-to-noise ratios, sensor–scalp distances, co-registration errors, and interfering internal noise sources on the classification performance.

## 2 Materials and Methods

Simulations were conducted using the development version of SPM (SPM12, release 6,https://github.com/spm/spm, downloaded 07/29/2022).

### 2.1 MRI acquisition and processing

A structural MRI scan was used to reconstruct the laminar surfaces that define the source space of our simulations and to inform the source reconstruction forward model. We employed quantitative multi-parameter mapping data (Carey et al., 2018;Weiskopf et al., 2013) from a single participant (male, 23 years) from the MEG UK database (https://meguk.ac.uk/database). Acquisition was performed on a 3T Prisma scanner equipped with a 32-channel receive radio frequency (RF) head coil (Siemens Healthineers, Erlangen, Germany) and a body RF receive coil at the Wellcome Centre for Human Neuroimaging, UCL, London. The study was approved by the University College London ethics committee (reference number 3090/001), and written informed consent was obtained from the participant prior to scanning. The high-resolution protocol was the same as inBonaiuto, Rossiter, et al. (2018), and consisted of three RF- and gradient-spoiled, multi-echo 3D FLASH scans with proton density-, relaxation time T1-, and magnetisation transfer-weighting (PDw, T1w, and MTw) at 800μm isotropic resolution, plus a map of the RF transmit field B1 acquired using a 3D-EPI spin echo/stimulated echo method (SE/STE) corrected for geometric distortions due to spatial inhomogeneities in the static magnetic field B0 (Lutti et al., 2010). For details on the acquisition protocol, seeEdwards et al. (2022).

Cortical surfaces were reconstructed using the recon-all pipeline from FreeSurfer (Fischl et al. (2004);https://surfer.nmr.mgh.harvard.edu). Because the contrast in the quantitative MRI maps deviates significantly from the T1w MPRAGE image contrast expected by the recon-all pipeline (Carey et al., 2018), the following steps were taken to extract an image with MPRAGE-like contrast from the 3T quantitative MRI parameters (seeMcColgan et al. (2021)). First, a small number of negative and very high values produced by estimation errors were set to zero in the longitudinal relaxation rate (R1) and PD maps, such that T1 (= 1/R1) was bounded between 0 and 8000 milliseconds (ms) and PD between 0 and 200%. Then, the PD and T1 maps were used as input to the FreeSurfer mri_synthesize routine to create a synthetic FLASH volume with optimal white matter (WM)/grey matter (GM) contrast (repetition time 20 ms, flip angle 30°, echo time 2.5 ms). This synthetic image was given as input to the SPM segment function (https://www.fil.ion.ucl.ac.uk/spm) to create a combined GM/WM/cerebrospinal fluid (CSF) brain mask (threshold: tissue probability > 0), which was used for skull stripping. The skull-stripped synthetic image then served as input for the remaining steps of the recon-all pipeline, which resulted in cortical surfaces for the GM/WM boundary (“white” surface), the pial surface, and the mid-cortical surface.

### 2.2 Simulated OPM-MEG sensor arrays and data

**Fig. 1.**
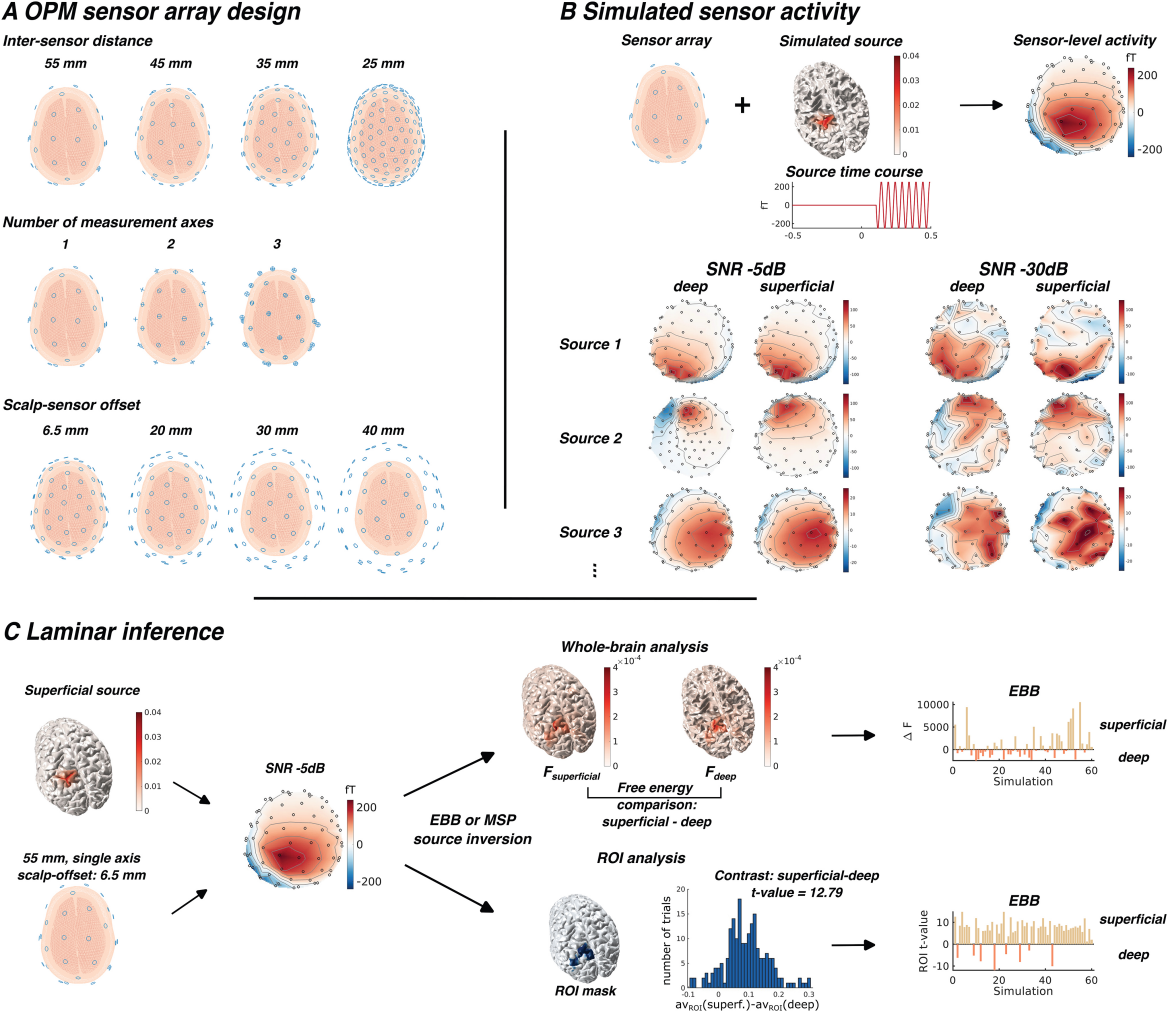
Simulation setup. (A) OPM sensor arrays were designed with varying sensor densities, measurement axes, and scalp–sensor offsets. (B) For each OPM array, we simulated dipolar current sources across 60 locations on each cortical surface. White noise was added to the sensor activity at different SNRs. The topoplots show the sensor activity averaged across trials for exemplary deep and superficial sources at SNRs of -5 and -30 dB. For the cortical surface plot, a patch size 10 times larger than in the simulations was used for better visualisation. The corresponding topoplots were based on the actual patch size used in the simulations (5 mm FWHM). (C) Laminar inference is demonstrated using a superficial source and an OPM array with 55 mm spacing, single-axis sensors, and a 6.5 mm scalp–sensor offset at -5 dB SNR. For the whole-brain analysis, source activity is separately reconstructed onto superficial and deep surfaces using either a Empirical Bayesian Beamformer (EBB) or Multiple Sparse Priors (MSP). The free energy difference between the two models indicates which surface is more likely. For the region-of-interest (ROI) analysis, source activity is reconstructed using a forward model with combined pial and white matter surfaces. An ROI is defined based on changes in activity from baseline. A paired t-test compares power changes at the superficial and deep surfaces, with the t-statistic indicating whether the superficial or deep surface shows greater activity. Laminar inference was repeated across 60 simulated sources on each surface for each OPM array design, SNR, and source reconstruction approach. The bar plots on the right show the differences in log model evidence and t-statistics.

We simulated single dipolar sources as measured by OPM-MEG sensor arrays of different configurations for all simulations. OPM datasets were generated with 200 trials, each lasting 1000 ms, at a sampling rate of 200 Hz using code adapted fromhttps://github.com/tierneytim/OPM(now integrated into SPM12). Sensor locations were determined using a point packing algorithm that positions sensors on the scalp surface at increasing densities as described previously (Tierney et al., 2020). We considered array designs with sampling distances between 25 and 55 mm in 10 mm increments, corresponding to 32, 42, 71, and 138 sensors for the single-axis array configurations (Fig. 1A, top row). Each sensor was modelled with one to three measurement axes: a single radial axis oriented normally to the scalp surface, a radial axis and a tangential axis, and a radial axis and two orthogonal tangential axes (Fig. 1A, middle row). With the orientation of the radial axis defined by the vector$\text{or}{\text{i}}_{r}=\left[x,y,z\right]$, the orientation of the first tangential axis is given by the vector$\text{or}{\text{i}}_{t_{1}}=\left[y,-x,0\right]$within the function spm_OPM_sim.m. The orientation of the second axis is orthogonal to the radial axis, as indicated by the dot product$\text{or}{\text{i}}_{r}\cdot \text{or}{\text{i}}_{t_{1}}$, which equals zero. The orientation of the second tangential axis is then defined by the cross product$\text{or}{\text{i}}_{t_{2}}=\text{or}{\text{i}}_{r}\times \text{or}{\text{i}}_{t_{1}}$.

The sensor–scalp offset was set to 6.5 mm, followingTierney et al. (2020). This corresponds to a typical distance between scalp and the centre of sensitivity of commercially available SERF OPM sensors (2nd generation QuSpin OPM sensors). We also simulated off-scalp MEG with increased scalp–sensor offsets of 20, 30, and 40 mm, by repositioning the sensors defined by the on-scalp OPM sensor array (scalp–sensor offset: 6.5 mm) along their radial axis to achieve the desired offsets (Fig. 1A, bottom row). Our aim was to compare laminar inference performance of OPM-MEG sensor arrays to conventional SQUID-based MEG, where sensors are placed further away from the scalp, and scalp–sensor offsets in the range of 15 to 40 mm are typical (Gu et al., 2021;Hill et al., 2020;Zetter et al., 2018,^2019^). Varying the scalp–sensor offsets also allowed us to evaluate the impact of possible design compromises concerning sensor placement in the OPM-MEG array, for example, due to the use of generic helmets (Hill et al., 2022) or the necessity to accommodate cooling pads in high-density sensor packaging in the case of alkali-based OPMs.

For each simulated OPM-MEG array, we then simulated deep and superficial current dipole sources at vertices on the white matter/grey matter boundary surface and the pial surface meshes, respectively. The meshes were constructed using the cortical surfaces (“white” and “pial”) derived from the FreeSurfer surface reconstruction pipeline applied to a participant’s structural MRI, as detailed inSection 2.1. The mesh resolution was determined by the pre-specified cortical meshes derived from the FreeSurfer surfaces, downsampled by a factor of 10. This resulted in 32,212 vertices for each surface, with an average vertex spacing of 1.74 mm for the white surface and 1.92 mm for the pial surface. Sources were positioned at mesh vertices, and the orientation of each cortical current source was defined by the surface normal of the cortical mesh at that location. The same meshes were later used for source reconstruction (seeSection 2.3).

For each surface, we randomly selected 60 vertices as cortical source locations. At each source location, a 20 Hz sinusoidal dipolar source patch with a patch size of 5 mm at Full Width at Half Maximum (FWHM) was added for each of the 200 trials modelled per cortical source location.

The spatial extent of a smooth Gaussian source patchqcentred at a vertex$v_{0}$is defined within SPM12 as follows:

$$q\left(i\right)=\{\begin{array}{cc} e^{-\frac{{\left(v_{i}-v_{0}\right)}^{2}}{2\sigma^{2}}}, & \text{if }\left(v_{i}-v_{0}\right)\leq \text{FWHM}\\ 0, & \text{otherwise} \end{array}$$ (1)

with the full width at half maximum FWHM defining the spatial extent of the activated patch,σ=FWHM/2.355describing the standard deviation that defines the smoothness of the current distribution, and${\left(v_{i}-v_{0}\right)}^{2}$the squared geodesic distance between vertex$v_{i}$and the patch centre vertex$v_{0}$.

The unit-less Gaussian patch values are normalised to sum up to 1 across the$N^{v}$vertices

$$q\left(i\right)=\frac{q\left(i\right)}{\sum_{j=1}^{N^{v}}q\left(j\right)}$$ (2)

and then multiplied with the sinusoidal current source to yield the current source density at vertex$v_{i}$and time t:

j(i,t)=q(i)j(t). (3)

The dipolar sources were active for 400 ms. Synthetic datasets were generated across a range of realistic SNRs by adding Gaussian white noise to the simulated data, scaled to yield per-trial amplitude SNR levels (averaged over all sensors), of -50, -40, -30, -20, -10, and -5 dB. The single-trial amplitude SNR in decibel is given by

$${\text{SNR}}_{\text{single\_trial}}=20{\text{log}}_{10}\left(\frac{A_{\text{signal}}}{A_{\text{noise}}}\right),$$ (4)

where$A_{\text{signal}}$is the signal amplitude and$A_{\text{noise}}$the noise amplitude.

We define the signal amplitude$A_{\text{signal}}$as the root mean square (RMS) signal strength, calculated as the mean of the standard deviations across channels for the sensor activity$D\in {\mathbb{R}}^{N_{\text{ch}}\times N_{\text{time}}}$, generated by the simulated current dipole source. For a given${\text{SNR}}_{\text{single\_trial}}$, the corresponding noise amplitude$A_{\text{noise}}$is obtained by rearrangingEquation 4:

$$A_{\text{noise}}=A_{\text{signal}}10^{-{\text{SNR}}_{\text{single\_trial}}/20}.$$ (5)

White Gaussian noise with amplitude$A_{\text{noise}}$was then added to the sensor dataDto achieve the desired single-trial SNR. Note that the SNR of the averaged sensor activity across trials is higher than that of a single trial. When averaging acrossNtrials, the signal amplitude increases linearly withN, while the noise amplitude increases with the square root ofN. For our simulations with 200 trials, this results in an SNR improvement over the single-trial SNR of

$$20{\text{log}}_{10}\left(\sqrt{N}\right)=20{\text{log}}_{10}\left(\sqrt{200}\right)\text{dB}\approx 23.01\text{dB.}$$ (6)

The SNR of the trial-averaged sensor data${\text{SNR}}_{\text{av}}$in our simulations is thus approximately 23 dB larger than the single-trial SNR:${\text{SNR}}_{\text{av}}\approx {\text{SNR}}_{\text{single\_trial}}+23.01\text{dB}$.

The forward model linking the simulated dipole current source to the sensor level activity was based on the Nolte single-shell approach, with the inner skull surface and cortical surfaces derived from the structural MRI. Sensors were assumed to be point magnetometers. The setup for simulating the OPM-MEG sensor activity is summarised inFigure 1B.

### 2.3 Laminar source estimation

We next aimed to determine the laminar origin of the simulated sources from the sensor data alone. We used two main types of analyses: a whole-brain analysis and an ROI-based analysis, equivalent to those described inBonaiuto, Rossiter, et al. (2018).

In the whole-brain analysis, we reconstruct the OPM-MEG sensor data once to the pial and once to the white matter cortical surface and then compare the fit of the two models using Bayesian model comparison as a metric (Bonaiuto, Rossiter, et al., 2018;Troebinger, López, Lutti, Bestmann, & Barnes, 2014). For Bayesian model comparison, we compute the difference in free energy between the pial and white matter forward models, approximating the log ratio of the model likelihoods. This results in a metric that is positive or negative, if there is more evidence for the pial or white matter model, respectively. A difference in log model evidence greater than 3 indicates that one model is approximately 20 times more likely than the other.

While this whole-brain analysis provides a global answer to the question of which cortical surface the sensor activity is more likely to originate from, it lacks spatial specificity regarding the location within the cortex where this laminar activity originates. To address this limitation, a region-of-interest (ROI) analysis that reconstructs the data onto both pial and white matter surfaces simultaneously can be employed (Bonaiuto, Rossiter, et al., 2018). An ROI is calculated based on the change of activity on either surface from a baseline time window, and the reconstructed activity within the ROI is compared between the two surfaces. We functionally define ROIs by comparing power in the 10–30 Hz frequency band during the period containing the simulated activity ([100 500] ms) with a prior baseline period ([-500 100] ms) at each vertex using two-tailed paired t-tests. Vertices in either surface with a t-statistic in the 75th percentile of the t-statistics over all vertices in that surface, as well as the corresponding vertices in the other surface, are included in the ROI. For each trial, we compute ROI values for the pial and white matter surfaces by averaging the absolute value of the change in power compared with baseline in that surface within the ROI. Finally, we use a paired t-test with variance regularisation (Ridgway et al., 2012) to compare the ROI values from the pial surface with those from the white matter surface over trials. The ROI analysis produces a t-statistic which is positive when the change in power is greater on the pial surface and negative when the change is greater on the white matter surface.

In both analyses, we estimated sources using the empirical Bayes beamformer (EBB;Belardinelli et al. (2012)andLópez et al. (2014)) and multiple sparse priors (MSP;Friston (2008)) source reconstruction approaches as implemented in SPM12 (https://www.fil.ion.ucl.ac.uk/spm/). The corresponding functional priors assume a sparse distribution of current flow across the cortex, uncorrelated in time for the EBB and locally coherent and sparse for the MSP approach. As inBonaiuto et al. (2018), for the whole brain analysis, the source inversion was applied to a Hann windowed time window from 500 ms to -500 ms filtered from 10 to 30 Hz, while no Hann window was used for the ROI analysis. These data were projected into 274 orthogonal spatial (lead field) modes and 4 temporal modes. Please note that projecting the time-domain sensor activity onto a smaller number of temporal modes—a standard preprocessing step before source inversion in SPM to enhance SNR and reduce computational burden—further increases the effective SNR. Cortical patches were modelled with a patch size of 5 mm FWHM. In this study, we used the development version of SPM as it calculates geodesic distances in the cortical mesh construction in an exact manner, while previous SPM implementations used the approximate Dijkstra algorithm.

We did not investigate simulations with minimum norm (MNE;Hämäläinen & Ilmoniemi (1994)) and LORETA (Pascual-Marqui et al., 1999) source localisation, as they have been shown in the past to be unable to allow laminar inferences for simulated sparse sources (Bonaiuto, Rossiter, et al., 2018). A replication of these findings can be seen inFigure S3in the Supplementary Material. We note that we simulated sparse sources and that our inability to distinguish between different laminar sources using the MNE or LORETA approaches can be at least partly attributed to the mismatch between data and functional prior assumptions.

The code for the current dipole sources simulations and the laminar inference was based on and adapted from the code athttps://github.com/jbonaiuto/laminar_sim.

### 2.4 Impact of co-registration errors

Laminar MEG using conventional SQUID-MEG relies on subject-specific head-casts to reach the required co-registration accuracy. To build the forward models, the accurate position and orientation of MEG sensors relative to the cortical surfaces derived from the MRI (i.e., co-registration of the two modalities) need to be established. Head-casts enable highly accurate co-registration in the sub-millimetre range and reduce head movements during scanning to less than 1 mm. This results in better data quality and anatomically more precise MEG recordings (Meyer et al., 2017;Troebinger, López, Lutti, Bradbury, et al., 2014) than for conventional co-registration strategies based on fiducials and surface mapping which typically achieve an accuracy of 5–10 mm (Friston, 2008;López et al., 2014) (but seeSonntag et al. (2018)who used an adaptive Metropolis algorithm to reach target registration errors between 1.3 and 2.3 mm at the head surface).

Accurate co-registration is also essential to fully utilise the high spatial resolution of on-scalp MEG systems. The expected co-registration error for OPM systems differs between flexible and more rigid sensor arrays (Iivanainen et al., 2019), with rigid helmets being preferred for high-resolution measurements due to their more accurate estimation of relative sensor locations and orientations (Hill et al., 2020). Here, we investigate the effect of co-registration errors expected for rigid sensor arrays by running an additional set of simulations. In these simulations, we added random displacement errors with a standard deviation ranging from 1 to 4 mm in 1 mm increments to each of the three fiducial locations in each direction before inverting the model. Introducing these fiducial errors changes the affine transformation matrix that aligns the MEG sensor array with the structural MRI, and, as a result, alters the position of the cortical sources (which are based on the MRI-derived cortical surface) relative to the MEG sensors. We used different random fiducial errors and recalculated the leadfield matrix for each of the 60 source locations within each set of simulations. Note that the impact of potential movements of the participant’s head in relation to the rigid sensor array during the experiment was not investigated in our study and needs to be further investigated.

### 2.5 Impact of source patch sizes

In the simulations described so far, we assumed cortical source patches with a width of 5 mm and used smoothed MSP and beamforming priors based on an equivalent smoothing kernel (as implemented in spm_eeg_invert_classic.m), that is, the estimated patch extent in our source reconstructions fitted the true simulated source patch extent. Previous simulation studies (Bonaiuto, Rossiter, et al., 2018;Troebinger, López, Lutti, Bestmann, & Barnes, 2014) have demonstrated that the under- or overestimation of patch extent can introduce biases in model evidence. Additionally, in experimental work (Bonaiuto, Meyer, et al., 2018), it was observed that smaller source prior patch sizes introduced a superficial bias, while larger patch sizes introduced a deep laminar bias. In line with previous studies (Bonaiuto, Rossiter, et al., 2018;Bonaiuto et al., 2021), the patch size in our main simulations was kept fixed to reduce the number of simulations required and to maintain conciseness. To address potential concerns regarding the misestimation of source extent, we conducted a set of additional simulations. We examined both congruent and incongruent patch sizes, using patch sizes of 5 and 10 mm, to investigate the impact of incongruent patch sizes on laminar inference performance with on-scalp OPM sensor arrays.

### 2.6 Impact of interfering brain sources

While simulations of single current dipoles can be illustrative, they do not reflect real experimental situations where laminar inference is performed on a source of interest in the presence of other interfering brain sources. To investigate the impact of such internal interfering sources, we simulated an additional toy example where we added five additional noise sources, similar toBoto et al. (2016)on the mid-cortical surface. The source time courses of the source of interest and the five internal noise sources were modelled as Gaussian random data within a frequency range of 10 to 30 Hz and the source amplitudes of the internal noise sources were defined in proportion to the source of interest with a relative source strength of 0.4.

In these simulations, the SNR of the simulated data with interfering brain noise sources varied across the 60 patch locations, as a different set of brain noise sources was selected for each simulation. On average, the noise amplitude of the brain noise sources,$A_{\text{noise\_brain}}$, defined as the root mean square (RMS) signal strength of the sensor data generated by solely the brain noise sources, yielded an average single-trial SNR of -18.24 dB (std: 5.25 dB) and an average SNR of -15.83 dB (std: 6.45 dB) for the trial-averaged data with added brain noise sources. Note that the improvement in trial-averaged SNR over the single-trial SNR is relatively small as, unlike white Gaussian sensor noise, the noise of the brain noise sources is coherent across trials. Given that for a single-trial SNR of -5 dB, the SNR of the trial-averaged data without added brain noise sources is approximately -5 dB + 23.01 dB = 18.01 dB, adding the interfering brain noise sources led to a drop in SNR of approximately 18.01 dB - (-15.83 dB) = 33.84 dB.

For an additional set of simulations with a larger number of interfering noise sources at a lower relative source strength of 0.1, refer to the Supplementary Material.

### 2.7 Alternative generative model (AGM)

In this work, we primarily use the same forward model, or generative model (GM), for both simulating data and performing source reconstruction, consistent with previous OPM-MEG (An et al., 2022;Boto et al., 2016;Mellor et al., 2024;Nugent et al., 2022;Tierney et al., 2020), as well as cryogenic MEG and/or EEG (e.g.,Allouch et al. (2023),Bonaiuto, Rossiter, et al. (2018),Hashemi et al. (2021), andO’Neill et al. (2021)) simulation studies. However, this approach has been criticised as committing an “inverse crime,” which can result in overly optimistic results (Wirgin, 2004).

Since selecting a specific alternative generative model (AGM) makes the conclusions heavily dependent on that particular model’s design, we chose to employ targeted manipulations of the forward model instead of selecting a specific AGM for all simulations. These manipulations, such as adding fiducial errors and adjusting patch sizes, provide greater interpretability.

To further explore the impact of using the same GM for both simulating and reconstructing sources, we also constructed an alternative generative model (AGM) for source simulation that captures some of the likely misestimations of the generative model. Building partially on the work ofHecker et al. (2021), we (1) changed the OPM sensor positions by adding random Gaussian distributed noise with a standard deviation of 1 mm to the x, y, and z coordinates and (2) adjusted the spacing of dipoles along the cortical surfaces by sub-sampling the original Freesurfer surfaces at a slightly larger factor than for the forward models used in the main manuscript. Specifically, the downsampled cortical surfaces for the AGM had approximately 11% of the original number of vertices in contrast to the 10% of the vertices for the forward model (the generative model GM) used in the main manuscript. This resulted in 35,433 dipole locations per surface in the source model of the AGM, which were placed at slightly different locations on the cortex compared with the source model of the GM with its 32,212 vertices per surface.

### 2.8 Statistics

We first compared the classification accuracy and bias of each analysis and source inversion algorithm by computing the percentage of sources that were classified correctly and the percentage of sources classified as coming from the pial surface and subjected these percentages to two-sided binomial tests with chance levels of 50% to evaluate their significance. To investigate whether these effects were also significant at the single-simulation level, we additionally employed a threshold of ±3 for the free energy difference (meaning that one model is approximately 20 times more likely than the other) and a threshold of the critical t-value with degrees of freedom (df) = 199 andα= 0.05 for the ROI t-statistic. By doing so we examined whether the classification approach not only found a difference in the correct direction, but also whether this metric was significant.

We used logistic regression to evaluate changes in classification accuracy and bias across sampling densities, number of axes, co-registration errors, and sensor–scalp offsets. Differences in laminar inference performance for free energy and the ROI-t-statistic analysis and for congruent and incongruent patch sizes were evaluated using exact McNemar’s tests (McNemar, 1947).

## 3 Results

### 3.1 At which signal-to-noise ratios can we draw laminar inferences?

We first evaluate the classification accuracy and bias across SNRs for an OPM-MEG array with a 35 mm inter-sensor distance to examine at which SNRs laminar inferences can be made using non-invasive OPM-MEG. We also report differences in laminar inference performance between the whole-brain free energy and the ROI-t-statistic analysis. Results for the single radial axis OPM sensor array with a 35 mm inter-sensor distance, corresponding to 42 sensors, are summarised inFigure 2.

**Fig. 2.**
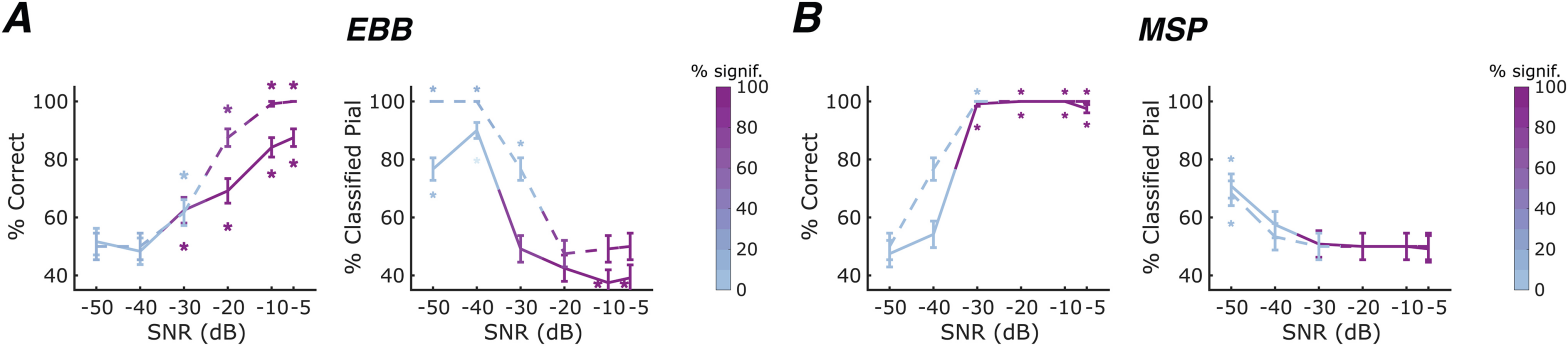
Laminar classification accuracy and bias across signal-to-noise ratios. We applied the (A) EBB and (B) MSP source reconstruction approaches to simulated data for an OPM-MEG sensor array with an inter-sensor distance of 35 mm. Solid lines represent the whole-brain free energy analysis, and dashed lines represent the ROI-t-statistic analysis. The left column within each sub-panel shows correct laminar inferences; the right column shows inferences favouring the pial source model. Line colour intensity indicates the percentage of simulations exceeding the significance threshold. Error bars represent standard error. Asterisks indicate significant deviations from chance levels. (A) For the EBB approach, we found significant increases in classification accuracy with increasing SNR. (B) For the MSP approach, we observed an excellent classification performance with accuracy at ceiling and no biases for SNRs of -30 dB or higher for both, free energy and ROI-t-statistic, analyses.

Classification accuracy improved with increasing SNR for both source reconstruction approaches and laminar inference analyses (EBB/free energy: beta = -1.168, p<0.001; EBB/ROI-t-statistic: beta = -2.038, p<0.001; MSP/free energy: beta = -2.941, p<0.001; MSP/ROI-t-statistic: beta = -3.341, p<0.001), while the percentage of sources classified as coming from the pial surface decreased with increasing SNR (EBB/free energy: beta = 1.292, p =<0.001; EBB/ROI-t-statistic: beta = 1.855, p<0.001; MSP/free energy: beta = 0.456, p<0.001; MSP/ROI-t-statistic: beta = 0.350, p<0.01). We note that for the EBB source reconstruction approach at high SNRs, classification was biased towards the deep surface when using the whole-brain analysis.

For the EBB source reconstruction approach, laminar source inference was statistically significant at SNRs of -20 dB or higher; however, we observed a bias towards the deep surface for the free energy metric. At these relatively high SNRs, the ROI-based analysis performed better than the whole-brain analysis: performance accuracy was higher (two-sided exact McNemar’s tests: p<0.001 at SNRs of -5, -10, and -20 dB) and classification less biased (two-sided exact McNemar’s tests: p<0.01 at SNRs of -5 and -10 dB). At an SNR of -30 dB, the ROI approach failed to yield significant results at the single-simulation level, that is, the absolute t-statistic values did not exceed the significance threshold. Additionally, laminar inference was biased towards the pial surface. While the free energy metric yielded a larger ratio of simulations with statistically significant classifications, classification accuracy was low. No laminar inference was possible at a very low SNRs of -40 and -50 dB for both analyses, with classification accuracy at chance level and a strong bias towards the pial surface.

The MSP approach yielded high classification performances and no significant biases at SNRs of -30 dB or higher. Note that there was a high accordance between the simulated data and the prior assumptions of the MSP approach, rendering this approach somewhat idealised. Classification performance did not differ significantly between the ROI-based analysis and the whole-brain analysis as shown by two-sided exact McNemar’s tests for classification accuracy and bias. At an SNR of -30 dB, however, the ROI approach failed to yield significant results reliably at the single-simulation level. At very low SNRs of -40 and -50 dB, classification accuracies and biases were not statistically significant at the single-simulation level for both, whole-brain and ROI-based, analyses.

In summary, we find that for an OPM-MEG sensor array with an inter-sensor distance of 35 mm, using the MSP approach (with patch priors that included the source locations) allowed us to achieve highly accurate laminar inferences at SNRs of -30 dB or higher. For the EBB approach, an SNR of at least -20 dB was required to reliably infer the correct laminar origin of simulated sources. Additionally, the ROI-based analysis is recommended over the whole-brain free energy analysis due to its superior classification accuracy and reduced bias.

Supplementary results for OPM sensor arrays with varying inter-sensor distances can be found in the Supplementary Material section inFigure S1. We also included a comparison between the results obtained using a forward model based on a three-shell boundary element model (BEM) and those obtained using the Nolte single-shell forward model, which was used throughout this study (Fig. S2). The Nolte single-shell model is commonly used in MEG source reconstruction and serves as the default model in SPM12 and Fieldtrip. While it has been demonstrated to exhibit competitive accuracy for MEG data (Stenroos et al., 2014), laminar inference may benefit from not only employing high-precision source spaces but also more accurate and anatomically realistic volume conductor models, based on Boundary Element or Finite Element Models, that take the different conductivities of head tissues into account. The results presented in the Supplementary Material highlight the potential for further improvement in laminar inference with a three-shell BEM model. Finally, we replicate previous results (Bonaiuto, Rossiter, et al., 2018) that we cannot successfully distinguish between superficial and deep sources using minimum norm and LORETA source localisation approaches (seeFig. S3).

### 3.2 Increasing sensor sampling density

We next investigated the impact of the sensor sampling density, here parameterised by the inter-sensor distance, on classification performance. Results are summarised inFigure 3. For the EBB approach combined with the whole-brain free energy analysis, classification accuracy significantly decreased with increasing inter-sensor distances at SNRs of -5 dB (beta = -0.710, p<0.01), -10 dB (beta = -0.781, p<0.001), and -20 dB (beta = -0.558, p<0.001), but not at lower SNRs, where laminar inference was challenging or not feasible at all. Sampling density had no discernible impact on classification bias, except at very low SNRs, where classification bias towards the white surface increased with increasing inter-sensor distances (-40 dB, beta = -1.398, p<0.001). However, single simulations were typically not significant and classification accuracy was at chance level.

**Fig. 3.**
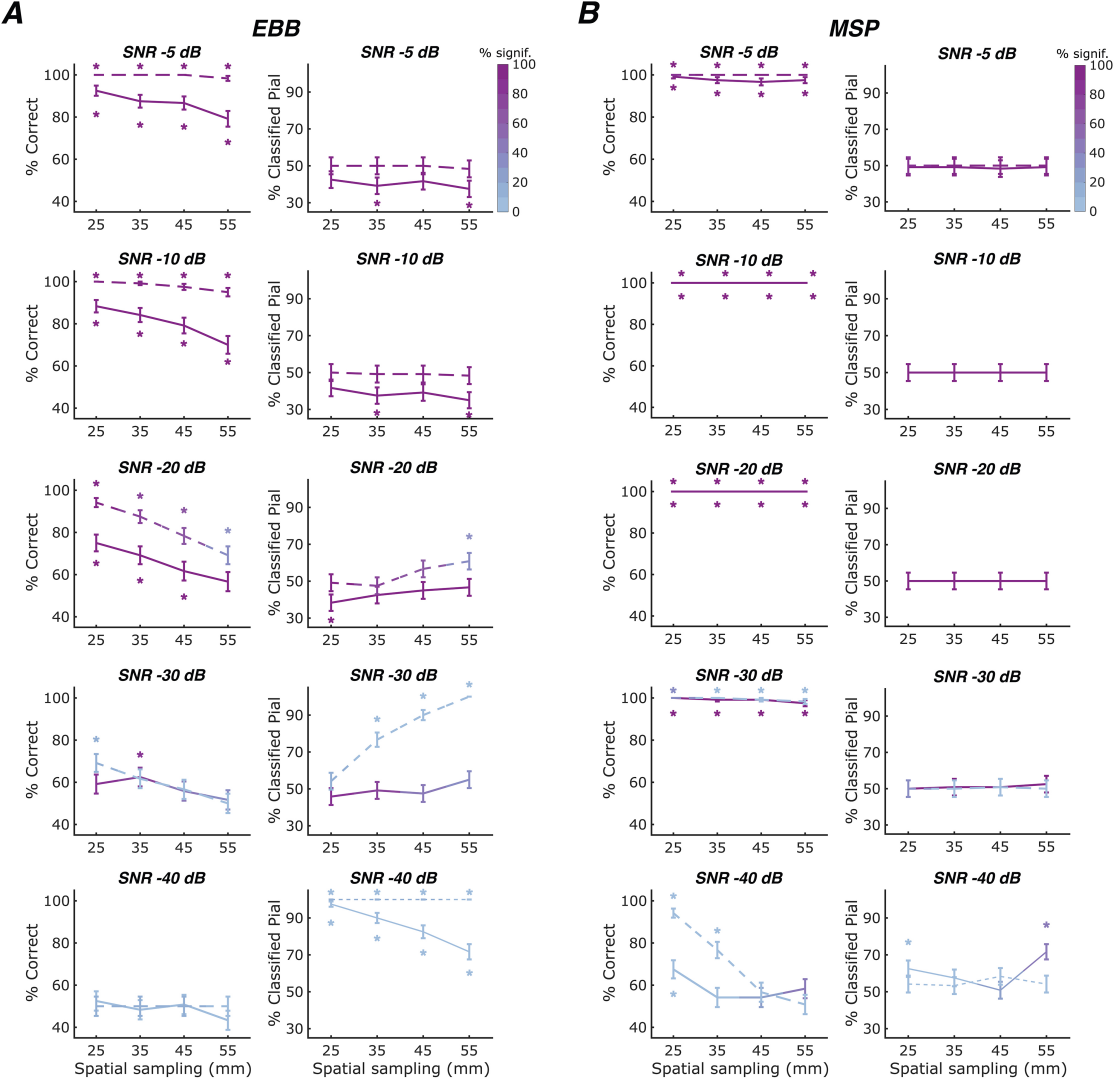
Laminar classification accuracy and bias across OPM array inter-sensor distances. Solid lines represent the whole-brain free energy analysis, and dashed lines represent the ROI-t-statistic analysis. The left column within each sub-panel shows correct laminar inferences; the right column shows inferences favouring the pial source model. Line colour intensity indicates the percentage of simulations exceeding the significance threshold. Error bars represent standard error. Asterisks indicate significant deviations from chance levels. SNR decreases across rows. (A) For the EBB approach, we found significant decreases in classification accuracy with increases in inter-sensor distances at SNRs of -20 dB or higher. (B) For the MSP approach, we observed no significant changes in laminar classification performance across sampling densities at SNRs of -30 dB or higher.

For the EBB approach combined with the ROI-t-statistics analysis, classification accuracy was at ceiling across sensor sampling densities at a high SNR of -5 dB and decreased significantly with increasing inter-sensor distances at SNRs of -10 dB (beta = -1.692, p<0.01), -20 dB (beta = -1.178, p<0.001) and -30 dB (beta = -0.522, p<0.01). Increasing inter-sensor distance led to a significant increase in bias towards the pial surface at -20 dB (beta = 0.356, p<0.05) and -30 dB (beta = 2.080, p<0.001). As described earlier, classification at the single-simulation level was reliably significant only for sensor array configurations with dense spatial samplings at -20 dB and not feasible at any spatial sampling densities at an SNR of -30 dB. At an SNR of -40 dB, the ROI-based t-statistic classification was extremely biased towards the pial surface across all sensor sampling densities. While the free energy metric was biased towards the pial surface as well, this bias decreased with decreasing sensor counts, that is, it was less pronounced at larger inter-sensor distances.

For the MSP approach, at SNRs of -30 dB or higher, classification performance was at ceiling and showed no significant biases. Classification performance and bias did not vary significantly across sampling densities. At these moderate-to-high SNRs, the whole-brain and ROI-based analyses yielded similar results as indicated by non-significant exact McNemar’s tests. At -30 dB, we observed a trend towards decreased classification accuracy with increasing inter-sensor distances for the free energy analysis (logistic regression: beta = -1.547, p = 0.082), while laminar inference tended to be non-significant at the single-source level for the ROI analysis.

At -40 dB, we observed a steep decline in performance accuracy with decreasing sensor counts (beta = -1.455, p<0.001). However, as mentioned earlier, the corresponding classifications were not reliably significant at the level of single simulations, that is, simulated sources.

Overall, in our simulations, we observed significant decreases in classification accuracy as the inter-sensor distances increased for the EBB source reconstruction approach. In contrast, the MSP approach demonstrated a consistent classification performance close to ceiling across varying inter-sensor distances at SNRs of -30 dB and higher.

### 3.3 Increasing the number of measurement axes

To investigate the impact of the number of measurement axes on laminar inference performance, we kept the sampling density fixed and varied the number of measurement axes. InFigure 4we report results for a simulated OPM-MEG array with 55 mm inter-sensor distance, which corresponds to 29, 58, and 87 channels for OPM arrays with single-axis, dual-axis, and triaxial sensors, respectively.

**Fig. 4.**
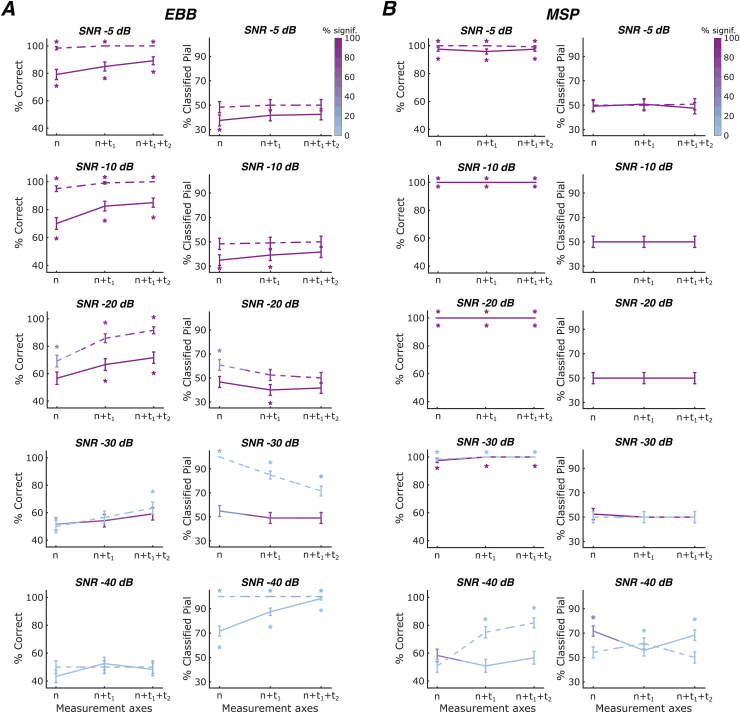
Laminar classification accuracy and bias across number of measurement axes for an OPM-MEG array with a sensor spacing of 55 mm. OPM sensor arrays were modelled with one to three measurement axes: a single radial axis oriented normally to the scalp surface (n), a radial axis and a tangential axis (n+t_1_), a radial axis and two orthogonal tangential axes (n+t_1_+t_2_). Solid lines represent the whole-brain free energy analysis, and dashed lines represent the ROI-t-statistic analysis. The left column within each subpanel shows correct laminar inferences; the right column shows inferences favouring the pial source model. Line colour intensity indicates the percentage of simulations exceeding the significance threshold. Error bars represent standard error. Asterisks indicate significant deviations from chance levels. SNR decreases across rows. (A) For the EBB approach, we found significant increases in classification accuracy with increases in the number of measurement axes at SNRs of -30 dB or higher. (B) For the MSP approach, both free energy and ROI-based analyses performed at ceiling for SNRs of -30 dB or higher, irrespective of the number of measurement axes.

We found that classification accuracy increased significantly with the number of measurement axes at SNRs of -20 dB or higher when using the EBB approach combined with the free energy analysis (SNR -5 dB: beta = -0.578, p<0.05; SNR -10 dB: beta = -0.709, p<0.01; SNR -20 dB: beta = -0.500, p<0.05). At these SNRs, we observed a bias towards the deep surface for the whole-brain analysis. This bias did not systematically increase or decrease with an increasing number of measurement axes. We found no advantage of increasing the number of measurement axes at SNRs of -30 dB and -40 dB. At -40 dB, we observed a bias towards the pial surface, which increased significantly with the number of measurement axes (beta = -1.767, p<0.001). Note that while this bias was strong, the underlying differences in free energy were not significant at the single-source level, that is, the absolute log free energy differences did not exceed the significance threshold of 3. For the ROI-based analysis, classification accuracy was close to ceiling for an SNR of -5 dB and increased with the number of measurement axes at -10, -20, and -30 dB (-10 dB: beta = -2.579, p<0.05; -20 dB: beta = -1.233, p<0.001; -30 dB: beta = -0.409, p<0.05). At an SNR of -30 dB, we observed a bias towards the pial surface which decreased significantly with added measurement axes (beta = 1.792, p<0.001). However, laminar inference was not statistically significant at the single-simulation level. At a very low SNR of -40 dB, classification accuracy was at chance level with an extreme bias towards the pial surface. Note that again these laminar inferences were not significant at the single-source level.

For the MSP approach, both whole-brain and ROI-based analyses performed near-ceiling for SNRs of -30 dB or higher, irrespectively of the number of measurement axes. At an SNR of -40 dB, classification accuracy increased strongly with the number of measurement axes for the ROI analysis (ROI: beta = -1.148, p<0.001); however, these laminar inferences did not exceed the significance threshold at the single-simulation level.

We anticipated that the advantage of a more homogeneous spatial coverage provided by sensors with multiple axes would be particularly evident for sparse OPM sensor arrays. Results indicating comparable yet less pronounced effects for a more densely arranged OPM sensor array can be found in theFigure S4.

In summary, our findings showed significant improvements in classification accuracy with an increasing number of measurement axes for the EBB source reconstruction approach. Conversely, the MSP approach exhibited classification performance close to ceiling levels for SNRs of -30 dB or higher, regardless of the number of measurement axes used.

### 3.4 Impact of increasing scalp–sensor offsets

To test for the impact of sensor–scalp offsets, we simulated an OPM sensor array with a 35 mm inter-sensor distance and radial single-axis configuration and increased the scalp–sensor offsets from 6.5 to 20, 30, and 40 mm. We expected a higher classification accuracy at sensors closer to the scalp, as being closer to the cortical surface (1) renders the ratio of the distances of the sensors to the deep and superficial surfaces larger and (2) results in larger leadfields, which have been linked to improved laminar inference performance (Bonaiuto, Rossiter, et al., 2018).

In contrast to our previous simulations, we determined the SNR here by adding fixed white Gaussian noise of varying magnitude (10, 30, 100, 300, and 1000 dB), rather than defining the SNR based on the sensor data. This approach was chosen to avoid masking the benefits of having the sensors closer to the brain sources. Defining the SNR based on the sensor data would have introduced relatively more noise due to the stronger sensor signals for sensors close to the scalp compared with those with a larger scalp–sensor offset.

**Fig. 5.**
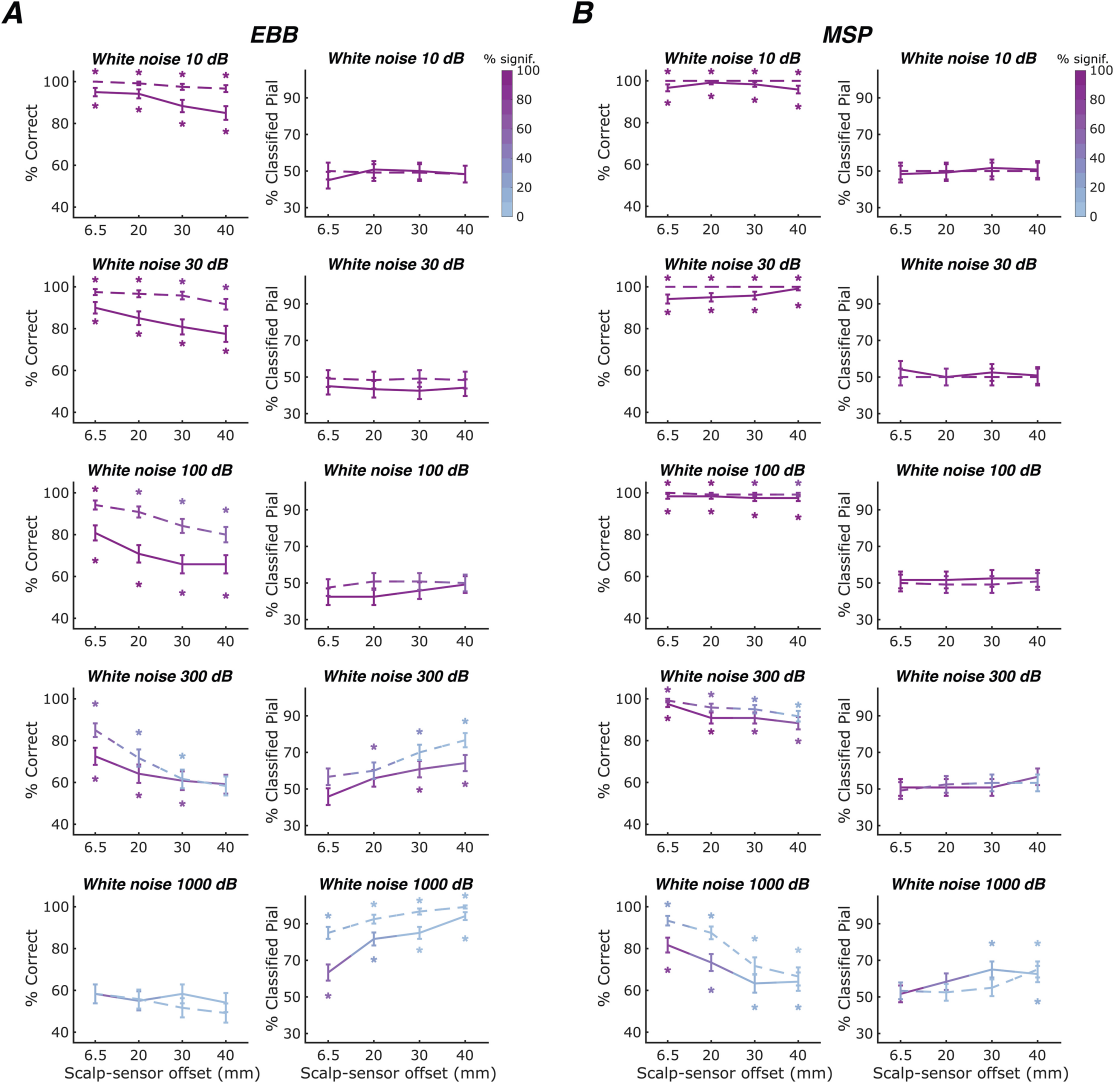
Laminar classification accuracy and bias across scalp–sensor offsets for an OPM-MEG array with a sensor spacing of 35 mm at the 6.5 mm scalp–sensor offset. Solid lines represent the whole-brain free energy analysis, and dashed lines represent the ROI-t-statistic analysis. The left column within each subpanel shows correct laminar inferences; the right column shows inferences favouring the pial source model. Line colour intensity indicates the percentage of simulations exceeding the significance threshold. Error bars represent standard error. Asterisks indicate significant deviations from chance levels. SNR decreases across rows. (A) For the EBB approach, increases in scalp–sensor distances led to decreasing laminar classification accuracy at added white noise of 300 dB or less in both analyses. At 300 dB of added white noise, classification bias towards the pial surface increased with larger scalp–sensor offsets, resulting in significant biases at greater offsets for both analyses. (B) For the MSP approach, classification accuracy remained at ceiling for added white noise levels of 100 dB or lower and did not vary significantly across scalp–sensor offsets. At noise levels of 300 or 1000 dB of added white noise, classification accuracy decreased significantly with increasing scalp–sensor offsets.

For the EBB approach, we found that increasing the scalp–sensor offset led to decreasing classification accuracy for added white noise of 300 dB or less for both analysis approaches (Fig. 5; 10 dB/whole-brain: beta = 0.856, p<0.01; 10 dB/ROI: beta = 1.365, p<0.05; 30 dB/whole-brain: beta = 0.596, p<0.01; 30 dB/ROI: beta = 0.891, p<0.05; 100 dB/whole-brain: beta = 0.477, p<0.01; 100 dB/ROI: beta = 0.889, p<0.001; 300 dB/whole-brain: beta = 0.375, p<0.05; 300 dB/ROI: beta = 0.843, p<0.001). At a noise level of 300 dB, this led to the classification accuracy no longer being significant at the largest tested scalp–sensor offset of 40 mm.

Classification bias did not change significantly across scalp–sensor offsets at noise levels of 100 dB added noise or less. At 300 dB added noise, classification bias towards the pial surface increased with increasing scalp–sensor offsets (whole-brain: beta = -0.494, p<0.01; ROI: beta = -0.626, p<0.001), becoming significant for offsets of 20 and 30 mm or larger for the ROI and whole-brain approaches, respectively. At the highest noise level of 1000 dB, the bias towards the pial surface also increased with increasing scalp–sensor offsets (whole-brain: beta = -1.316, p<0.001; ROI: beta = -1.575, p<0.001). However, classification accuracy did not differ significantly from chance level at this noise level, regardless of the scalp–sensor offset.

For the MSP source reconstruction approach, classification accuracy was high at noise levels of 10, 30, and 100 dB added noise for both the whole-brain and the ROI-based analyses, with neither classification accuracy nor bias varying significantly across scalp–sensor offsets. At a noise level of 300 dB, classification accuracy decreased significantly with increasing scalp–sensor offsets (whole-brain: beta = 0.704, p<0.05; ROI: 1.056, p<0.01), while classification bias did not vary significantly across scalp–sensor offsets. At the highest noise level of 1000 dB, classification accuracy again decreased significantly with increasing scalp–sensor offsets (whole-brain: beta = 0.597, p = 0.001; ROI: 1.201, p<0.001). Additionally, we observed a significant increase in bias towards the pial surface with increasing scalp–sensor offsets for the whole-brain approach (beta = -0.328, p<0.05). For the ROI-based approach, this increase was only a trend (beta = -0.304, p = 0.065).

In summary, increasing scalp–sensor distances reduced laminar classification accuracy for the EBB source reconstruction approach at noise levels of 300 dB or lower, with an increasing bias towards the pial surface at 300 dB. The MSP approach maintained high accuracy at noise levels of 100 dB or lower, but accuracy decreased significantly with increasing scalp–sensor offsets at 300 and 1000 dB.

### 3.5 Co-registration errors

**Fig. 6.**
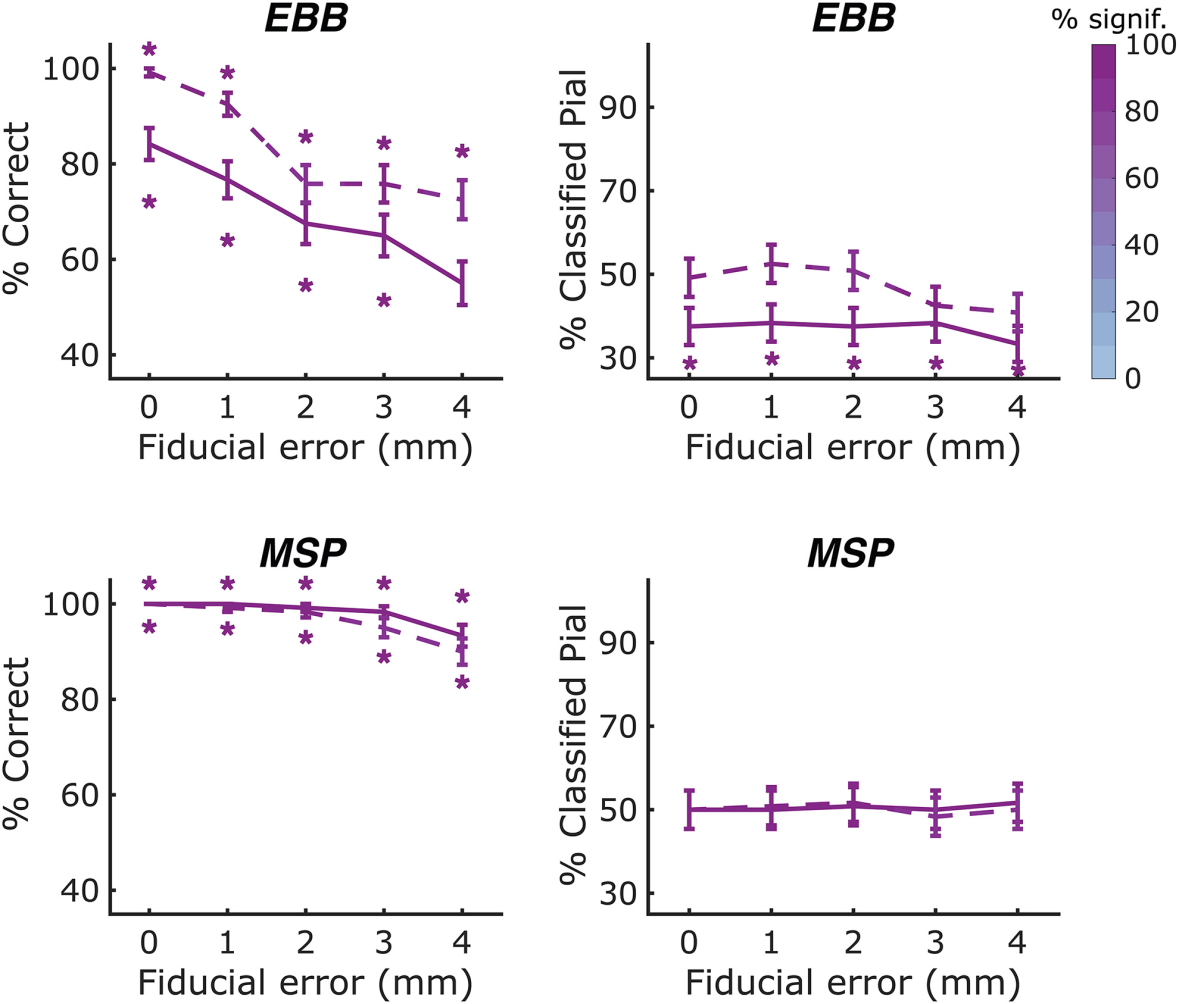
Laminar classification accuracy and bias across co-registration errors at an SNR of -10 dB. We used an OPM-MEG array with a sensor spacing of 35 mm and applied the EBB and MSP source reconstruction approaches to the simulated data. Co-registration errors were operationalised by adding random displacement errors with a standard deviation from 1 to 4 mm in 1 mm increments to each of the three fiducial locations in each direction. Solid lines represent the whole-brain free energy analysis, and dashed lines represent the ROI-t-statistic analysis. The left column shows correct laminar inferences; the right column shows inferences favouring the pial source model. Line colour intensity indicates the percentage of simulations exceeding the significance threshold. Error bars represent standard error. Asterisks indicate significant deviations from chance levels. For the EBB approach, we observed a decrease in classification accuracy with increasing co-registration errors. We did not observe any significant changes in classification bias with varying co-registration errors for either the whole-brain or the ROI-based analysis. For the MSP approach, classification accuracy decreased significantly with increasing co-registration errors. No classification biases were observed for either the whole-brain or ROI-based analysis.

To investigate the impact of co-registration errors on our ability to perform non-invasive laminar inference, we ran simulations with a single-axis array at an inter-sensor distance of 35 mm and an SNR of -10 dB and added small random displacements to the three fiducial locations (Fig. 6). We observed a steep decrease in classification accuracy with increasing co-registration errors for the EBB approach (whole-brain: beta = 0.838, p<0.001; ROI: beta = 1.189, p<0.001), where laminar inference was not feasible anymore at a fiducial displacement error of 4 mm. Classification bias remained relatively stable across co-registration errors, with a trend towards an increased bias towards the deep surface with increasing co-registration errors for the ROI-based t-statistics analysis (beta = 0.268, p = 0.065). For the free energy analysis, the previously observed bias towards the deep surface was sustained across co-registration errors.

For the MSP approach, classification accuracy remained high (above 90%) across co-registration errors, but decreased significantly with increasing co-registration errors (whole-brain: beta = 2.497, p<0.001; ROI: beta = 1.908, p<0.01). No classification biases were observed for either the whole-brain or the ROI-based analysis.

### 3.6 Incongruent patch sizes

To test for the impact of incongruencies between the true and the assumed source extent, we simulated congruent and incongruent source and reconstruction patch sizes of 5 and 10 mm for a single-axis OPM-sensor array with an inter-sensor distance of 35 mm and performed laminar inference using the whole-brain free energy analysis.

**Fig. 7.**
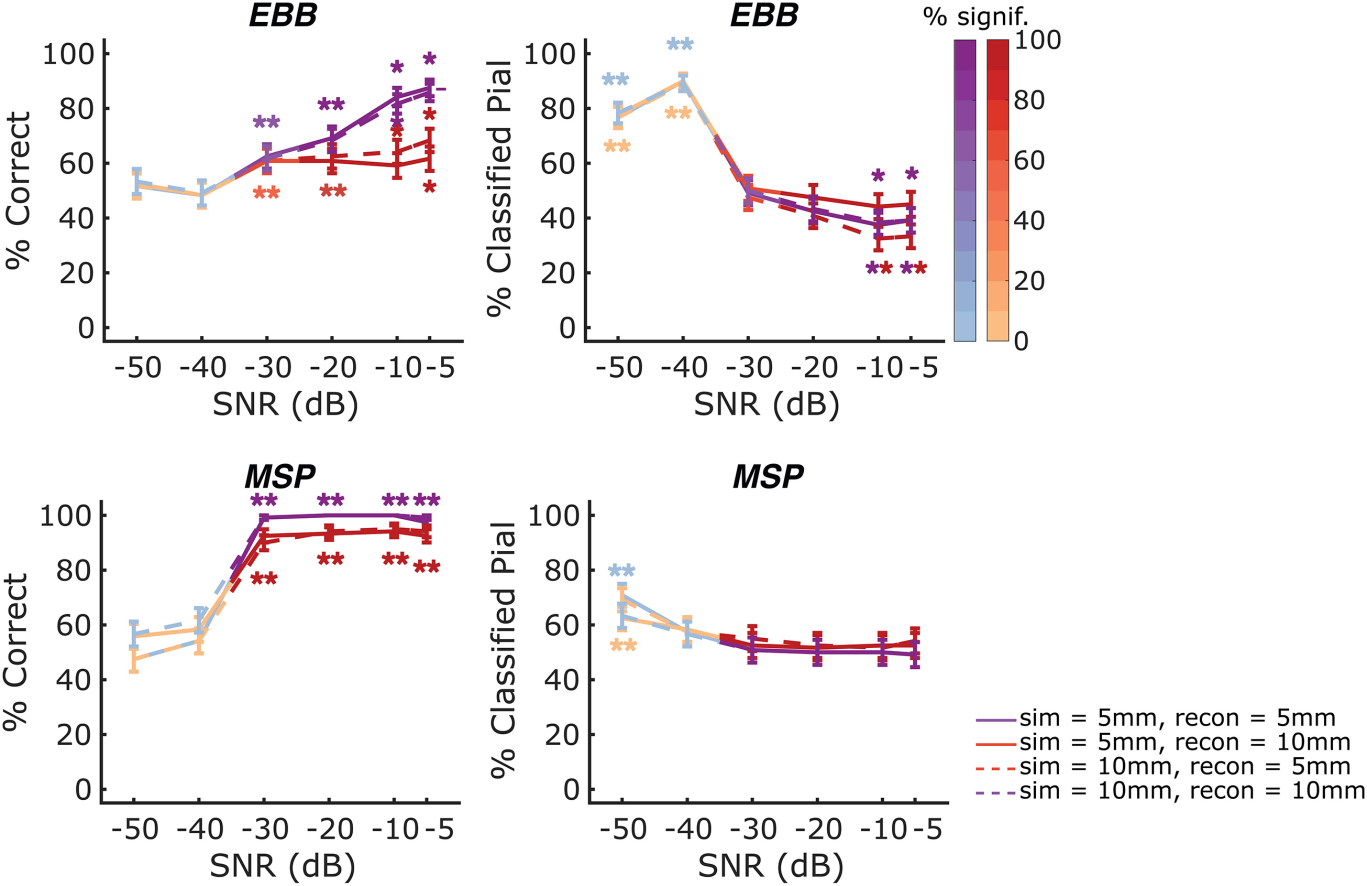
Whole-brain free energy analysis laminar inference with congruent and incongruent patch sizes for an OPM-MEG array with a sensor spacing of 35 mm. Purple lines denote simulations where the reconstructed patch size matches the simulated patch size (solid = 5 mm, dashed = 10 mm), red lines are where patch size is either over- (solid) or underestimated (dashed). The left column shows correct laminar inferences; the right column shows inferences favouring the pial source model. Line colour intensity indicates the percentage of simulations exceeding the significance threshold. Error bars represent standard error. Asterisks indicate significant deviations from chance levels. As SNR increased, classification accuracy was reduced for both source reconstruction approaches when the patch size was under- or overestimated. With increasing SNR, congruent patch sizes resulted in a bias towards the deep surface for the EBB approach. Overestimation of patch sizes reduced this bias, while underestimation of patch sizes led to an even stronger bias towards the deep surface. The MSP approach showed no bias as SNR increased, irrespective of whether congruent or incongruent patch sizes were used.

For the EBB source reconstruction approach, we found a significant drop in laminar classification accuracy for over- (at SNRs of -5, -10, and -20 dB: p<0.01, p<0.01 and p<0.05, respectively) and underestimated (at SNRs of -5 and -10 dB: both p<0.00001) patch sizes (Fig. 7). At SNRs of -5 dB and -10 dB, simulations with congruent patch sizes (shown in purple) were biased towards deep layers as observed in our previous simulations (Figs. 2-4). Compared with congruent patch sizes, overestimation of patch sizes yielded a shift towards pial classification (rendering the classification bias non-significant), while underestimation of patch sizes led to an even stronger bias towards the deep cortical surface. However, two-sided exact McNemar’s tests indicated that these shifts were not statistically significant at any of the SNRs tested.

For the MSP approach, we found smaller but significant decreases in classification accuracy for incongruent patch sizes (overestimated patch sizes at SNRs -10, -20, and -30 dB: p<0.05, p<0.01 and p<0.05; underestimated patch sizes at SNRs of -20 and -30 dB: p<0.05 and p<0.001, respectively) and no classification bias for neither congruent or incongruent patch sizes at all SNRs that enable statistically significant laminar inference (-30 dB or higher).

### 3.7 Interfering brain noise sources

Next, we investigated the impact of internal noise sources on our ability to accurately infer the laminar origin of the simulated sources. To this end, we modelled sensor activity from a laminar cortical source of interest at an SNR of -5 dB and added concurrent weaker noise sources on the mid-cortical surface. We simulated the sensor activity for a dense OPM-MEG array with an inter-sensor distance of 25 mm and single-axis sensors and again applied the whole-brain and ROI-based laminar inference analyses for EBB and MSP source reconstruction approaches. Results are summarised inFigure 8.

**Fig. 8.**
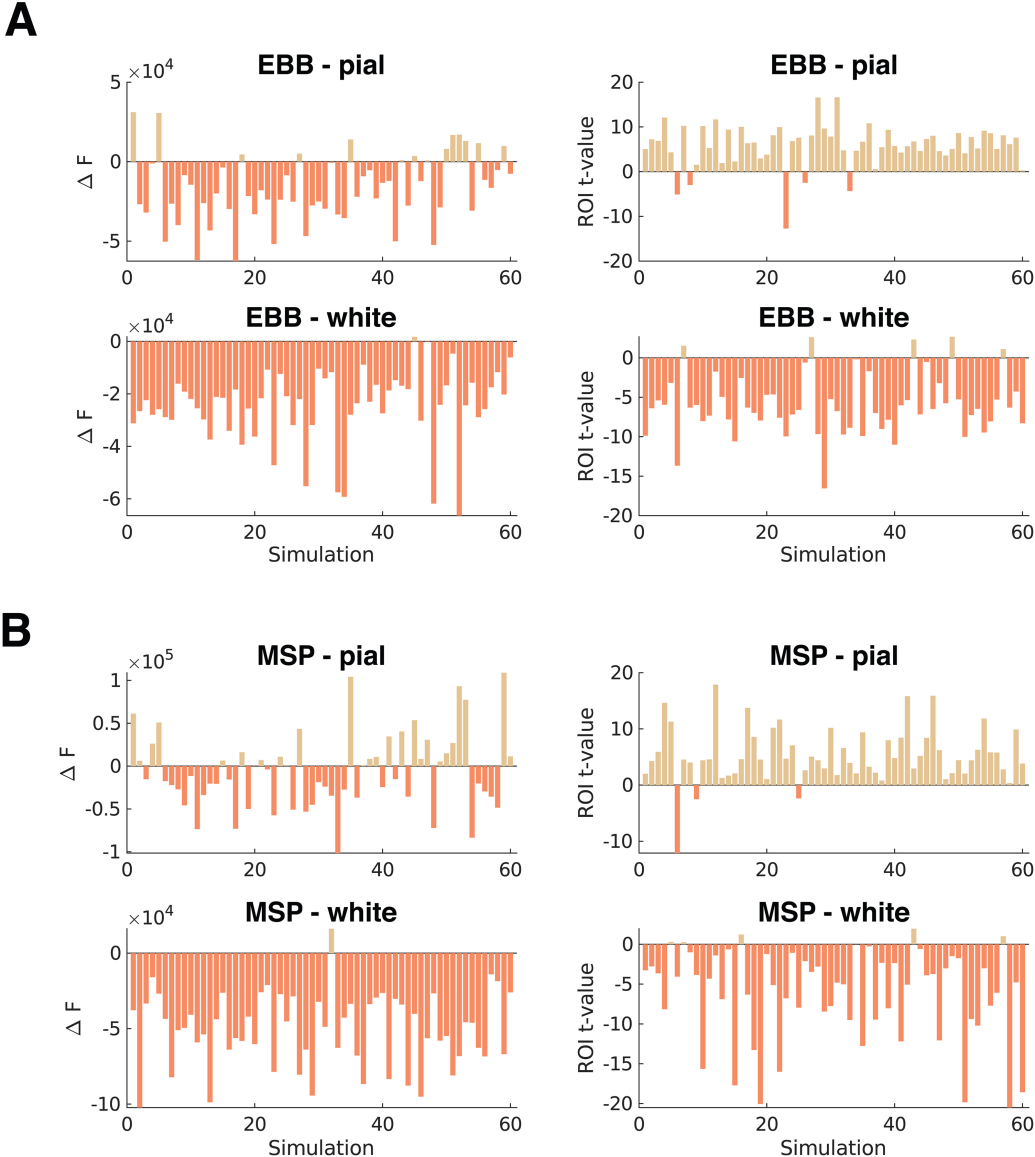
Laminar source discrimination in the presence of interfering brain noise sources. We used a dense OPM-MEG array with an inter-sensor distance of 25 mm and simulated sensor activity at a relatively high SNR of -5 dB to mimic a best-case scenario. Left column: The difference in free energy between the pial and white matter generative models in each simulation. Right column: t-values for the difference between pial and white matter ROI values for each simulation. Each panel shows simulations with pial surface sources on the top row, and simulations with white matter surface sources on the bottom row. For the (A) EBB and (B) MSP source reconstruction approach, the whole-brain analysis similarly showed a low classification accuracy and a bias towards the deep surface, while the ROI-based analyses yielded high classification accuracies and showed no classification bias.

For the EBB source reconstruction approach and the whole-brain analysis, we were not able to infer the laminar origin of the simulated sources (correct = 54.17%, p = n.s.) and observed a strong bias to the deep surface (white matter = 87.50%, p<0.00001). This contrasts with our results for the ROI analysis, which showed a high classification accuracy (EBB: correct = 91.67%, p<0.00001) and no bias (pial = 50.00%, n.s.). For the MSP source reconstruction approach, the whole-brain analysis similarly showed a low classification accuracy, which, however, was still above chance level (correct = 60.83%, p<0.05), and laminar inference was biased towards the deep surface (white matter = 76.67%, p<0.0001). For the ROI analysis, the MSP approach yielded a high classification accuracy (correct = 93.33%, p<0.00001) and showed no classification bias (pial = 51.67%, n.s.).

### 3.8 Alternative generative model (AGM)

We next investigated the impact of using different forward models for data generation and source reconstruction using an AGM with slightly adapted OPM sensor positions and spacing of dipoles along the cortical surfaces. Results are summarised inFigure 9. For the EBB source reconstruction approach at high SNRs, laminar inference accuracy was lower when using the AGM for simulations than when the same GM was used for both data generation and inversion (two-sided exact McNemar’s tests, whole-brain: p < 0.05 at an SNR of -5 dB; ROI: p<0.05 at SNRs of -5 and -10 dB). However, overall classification performance was relatively comparable. For the MSP approach, the whole-brain analysis showed a marked decrease in laminar inference accuracy (two-sided exact McNemar’s tests, whole-brain: p<0.0001 for SNRs of -30 dB or higher), with accuracy at chance level for all SNRs except the highest SNR of -5dB. We hypothesise that the sharp decrease in classification accuracy under the AGM model arises from the prior patches used in MSP source inversion no longer aligning with the actual source locations. This misalignment likely reduces overall model evidence and poses additional challenges for laminar inference. Remarkably, classification performance for the ROI-based analysis was not affected by using the AGM instead of the GM for source generation.

**Fig. 9.**
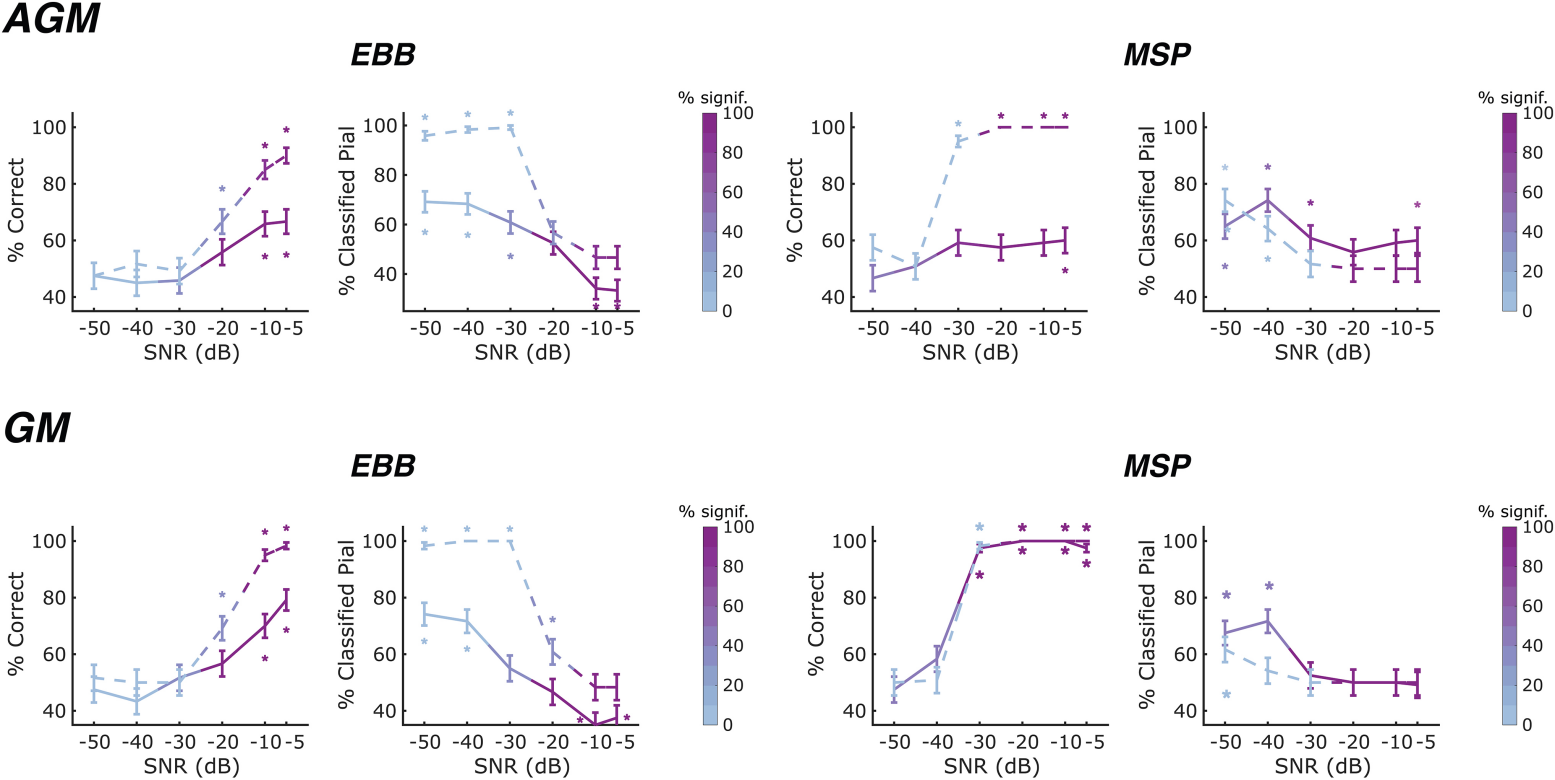
Laminar inference using an alternative generative model (AGM) for source generation. We constructed an alternative forward model (AGM) with slightly altered sensor locations and dipole locations on the cortical surfaces for source simulation and compared its performance with the forward (or generative) model (GM) used for both MEG data generation and reconstruction in the simulations in the main manuscript. Simulations were performed for an OPM sensor array with an inter-sensor distance of 55 mm and single-axis sensors. Solid lines denote laminar inference based on the whole-brain free energy analysis; dashed lines denote laminar inference based on the ROI-t-statistic analysis. The left column shows the percentage of correct laminar inferences, the right column shows the percentage of simulations where laminar inference favoured the pial source model. The percentage of simulations with free energy differences or t-statistics exceeding the significance threshold is represented by the intensity of the line colour. The error bars represent the standard error. Asterisks show where the percentage is significantly above or below chance levels. For the EBB source reconstruction approach, laminar inference performance was slightly lower when using the AGM compared with simulations where the same forward model (GM) was used for both MEG data generation and reconstruction at high SNRs, but overall, the performance was otherwise comparable. For the MSP source reconstruction approach, we observed a dramatic decrease in classification accuracy for the whole-brain analysis, while no significant differences in laminar inference were observed between the AGM and GM simulations for the ROI-based analysis.

## 4 Discussion

We evaluated the efficacy of forward models with a high-precision cortical source space consisting of deep and superficial cortical surfaces (Bonaiuto, Rossiter, et al., 2018;Meyer et al., 2017;Troebinger, López, Lutti, Bestmann, & Barnes, 2014;Troebinger, López, Lutti, Bradbury, et al., 2014) in achieving laminar discrimination using on-scalp OPM sensors. We find that adequate laminar inference can be achieved with relatively few sensors at coarse spatial samplings (>50 mm) and moderate-to-high SNRs and demonstrate the benefit of positioning the sensors closer to the scalp. For the EBB approach, classification accuracy improved with increasing spatial sampling densities and measurement axes. For the MSP approach, classification performance was near-ceiling for SNRs of -30dB or higher and unattainable at lower SNRs, regardless of the spatial sampling employed.

As expected, we observed that laminar inference was vulnerable to over- and underestimation of source patch sizes, co-registration errors, and interfering brain sources. The biases observed in laminar inference also warrant caution. We found strong biases towards both the superficial and deep surfaces, particularly at very low SNRs, although these biases did not typically reach statistical significance. Furthermore, the EBB approach, when combined with whole-head free energy analysis, exhibited a notable bias towards the deep surface, particularly at high SNRs. Since at high SNRs, beamformer images tend to be more focal (Hillebrand & Barnes, 2003), we propose that the denser sampling of the deep surface could be advantageous in such scenarios.

Based on our findings, we recommend using either the MSP approach or adopting the ROI-based analysis for the EBB approach, as it demonstrated higher classification accuracy than the whole brain analysis without exhibiting a bias towards the white surface. However, we note that the excellent performance of the MSP approach at SNRs above -30 dB can be partially attributed to the distinct advantage of its source space priors, which include the patches where sources were simulated.Figure 9provides evidence that such an idealised setting can lead to overly optimistic results. In these additional simulations, we use a slightly modified alternative forward or generative model (AGM) for data generation and thereby avoid the “inverse crime” of using the same source space for generating and reconstructing the data (Wirgin, 2004). Notably, while the whole-brain analysis was significantly impacted by using an AGM instead of the same forward model for both data generation and reconstruction, the classification performance for the ROI-based analysis remained unaffected.

Regardless of the chosen approach, ensuring adequate SNR through an appropriate number of trials and shielding, as well as maintaining precise co-registration accuracy, is crucial. Bayesian model comparison across various patch sizes can assist in the decision-making process of selecting a suitable patch size (Bonaiuto, Meyer, et al., 2018). In the following section, we discuss the results in more detail.

### 4.1 Number of sensors

As expected, increasing spatial sampling density led to increased classification accuracy, in line with previous studies that have shown increased spatial resolution for source-reconstructed activity with increasing sensor counts for SQUID-MEG (Vrba et al., 2004) and OPM-MEG (Boto et al., 2016;Clancy et al., 2021;Iivanainen et al., 2017;Nugent et al., 2022). However, our finding that laminar inference is possible at a low sensor count of 32 sensors was somewhat surprising given that previous studies have suggested that approximately 300 sensors are needed to achieve a spatial discrimination of 2 to 2.5 mm (Boto et al., 2016;Tierney et al., 2020). We argue that employing forward models with high-precision source spaces—defined by the deep and superficial cortical surfaces—enabled us to exploit the small differences in leadfields between the two source surfaces and thus effectively enhanced the spatial resolution.

### 4.2 Number of measurement axes

Classification accuracy increased with the number of measurement axes, consistent with findings that increasing the number of axes results in increased information content (Iivanainen et al., 2017) and better spatial coverage (Boto et al., 2022;Brookes et al., 2021). As expected, the benefit of multiple axes was more pronounced at lower inter-sensor distances, where undersampling of higher spatial frequency features leads to unexplained noise due to signal aliasing. Note that our simulations did not include external noise sources, and the advantage of dual-axis and triaxial sensors is likely to be even greater in the presence of such interference (Brookes et al., 2021).

### 4.3 Impact of sensor–scalp offset distance

One of the fundamental assumptions behind the postulated potential of OPMs for non-invasive electrophysiology is that sensors closer to the scalp will increase the sensitivity to cortical sources, resulting in higher information capacities and spatial resolution as well as better source separability compared with conventional MEG (Iivanainen et al., 2017;Riaz et al., 2017;Tierney et al., 2020). Our simulations indicate that decreasing the scalp–sensor offset indeed increases classification accuracy. In some instances, specifically at a noise level of 300 dB added Gaussian noise for the EBB approach and at a noise level of 1000 dB added Gaussian noise for the MSP approach, the scalp–sensor offset was decisive in whether classification accuracy was above chance level or free from significant classification bias, emphasising the potential of on-scalp sensors such as OPMs for improving the accuracy and reliability of laminar inference.

### 4.4 Impact of co-registration errors

Classification accuracy decreased with increasing co-registration errors for both source reconstruction approaches. However, laminar inference remained possible for fiducial errors of up to 4 mm, depending on SNR, sensor density, as well as the source reconstruction approach and laminar inference analysis used.

Here we modelled co-registration errors for a rigid sensor array, that is, we assume systematic shifts due to fiducial localisation errors like in SQUID-MEG, rather than random sensor localisation or orientation errors. The latter can have a more detrimental effect on source reconstruction accuracy (Hill et al., 2020).Zetter et al. (2018), using random displacement errors, proposed a cut-off of 4 mm sensor position and 10° sensor orientation RMS errors for acceptable mis-co-registration, noting that at larger co-registration errors, the advantage of on-scalp MEG may be lost.Troebinger, López, Lutti, Bestmann, and Barnes (2014), in a simulation study based on a SQUID-MEG rigid helmet, added rotation or pure translation to fiducial locations and suggested a cut-off of less than 2 mm/2° of fiducial error for accurate laminar inferences. Localising sensors with this degree of accuracy is challenging but feasible, as shown for co-registration between on-scalp MEG and MR images (Cao et al., 2021;Zetter et al., 2019). Even higher accuracy, with errors below 0.5 mm, may be necessary for localising multiple, dependent sources with added noise sources (Nugent et al., 2022). For experimental setups, the use of rigid measurement helmets in conjunction with co-registration devices with an excellent accuracy, such as structured-light or laser scanners, will be critical to reach the necessary co-registration accuracies.

Co-registration errors are not the only errors that will affect forward model accuracy and, consequently, localisation accuracy. It is worth noting that we did not evaluate the impact of crosstalk and gain errors (Duque-Muñoz et al., 2019), orientation errors, or cross-axis projection errors (Borna et al., 2022;Iivanainen et al., 2019;Robinson et al., 2022).

### 4.5 Patch size incongruencies

We have replicated previous findings that over- or underestimation of patch sizes can decrease classification accuracy and bias laminar results (Bonaiuto, Rossiter, et al., 2018;Troebinger, López, Lutti, Bestmann, & Barnes, 2014). Compared with congruent patch sizes, overestimation of patch sizes resulted in a shift towards pial classification, rendering the classification bias non-significant, while underestimation of patch sizes led to an even stronger bias towards the deep surface. We note that these shifts in bias with incongruent patch sizes were not statistically significant at any SNR as evaluated with the exact McNemar’s test. However, these results are consistent with earlier findings, and we interpret them as follows: larger patch sizes and deeper sources both lead to spatially more spread-out sensor signals, and underestimation of patch sizes and the resulting unexpected lower spatial frequencies at the sensor level can be explained by assigning a source to the deep cortical surface. Similarly, a sensor topography more focal than expected from overestimated patch sizes can be explained by assigning a source to the superficial surface (Troebinger, López, Lutti, Bestmann, & Barnes, 2014). Even though the ground truth regarding the source extent is unknown for experimental data, the optimal patch size for the laminar inference procedure can be determined by comparing the model evidence across varying reconstruction patch sizes for a forward model with combined deep and superficial surfaces (Bonaiuto, Meyer, et al., 2018).

### 4.6 Interfering brain noise sources

We next investigated laminar classification performance in the presence of five interfering brain noise sources on the mid-cortical surface. We observed that, despite employing a favourable setup with high SNR and dense sensor sampling, the whole-brain analysis was unable to successfully recover the laminar origin of the source of interest. Performance accuracy of the ROI-based approach remained high and did not show any bias in the presence of noise sources. We note that the laminar source of interest had a larger SNR than the interfering noise sources, and the activity-based ROI was thus mostly defined by the source of interest, effectively masking the noise sources. We further highlight that while we modelled the interfering brain noise sources at the mid-cortical layer, no surface for this mid-cortical layer was incorporated into the source space of the forward model. Such forward models with a more fine-grained laminar resolution offer an exciting perspective for future studies.

### 4.7 Alternative approaches to infer laminar MEG activity

As we conclude this paper, we would like to highlight some alternative approaches to inferring the laminar origin of simulated current dipoles. While we were able to successfully infer the laminar origins of simulated current dipoles using OPM-MEG sensor arrays at relatively low spatial sampling densities, other methods have also been proposed.

Pinotsis et al. (2017)andPinotsis and Miller (2020)employed dynamical causal modelling (DCM), with modelling parameters set based on estimates from intracranial data, and statistical decision theory to infer the laminar sources of non-invasive electrophysiological signals. However, the authors note that applying laminar DCM to non-invasive data is challenging due to the high collinearity of these parameters, and their results could not be replicated across datasets (Pinotsis & Miller, 2020). In a proof-of-principle study,Ihle et al. (2020)combined DCM and a nested laminar forward model to recover the laminar origin of a cortical current source. Yet, their approach was restricted to a single dipole pair at a known spatial location, limiting their applicability to more realistic scenarios.

A promising path towards non-invasive laminar inference could be to combine classical dipole fitting or beamformer source reconstruction with high-density OPM arrays. Recent simulation work (Nugent et al., 2022) estimated that a densely packed magnetocorticography array of 56 OPM sensors would be able to localise multiple electrophysiological brain responses at a millimetre resolution.

## 5 Conclusion

We conducted simulations to investigate the potential of on-scalp OPM sensors combined with high-precision source space modelling for inferring the laminar origins of neural activity. Our findings demonstrate the advantage of positioning the sensors closer to the scalp and indicate that OPM arrays can achieve laminar inference with relatively low sensor counts and moderate-to-high SNRs. These results provide guidance on the requirements for OPM-MEG systems to achieve effective laminar source localisation.

## Supplementary Material

Supplementary Material

## Data Availability

The scripts and materials for the simulations and analyses reported in this manuscript will be made openly available on GitHub (https://github.com/sashel/laminar_sim_opm).
