# Supplementary material for: Inferring laminar origins of MEG signals with optically pumped magnetometers (OPMs): A simulation study
Source: Imaging Neurosci (Camb). 2025 Jan 2;3:imag_a_00410. doi: 10.1162/imag_a_00410 (PMC12319968; doi:10.1162/imag_a_00410)
Supplement: Supplementary Material [file imag_a_00410-supp.pdf]

Supplementary material

Laminar inference across SNRs at varying inter-sensor distances

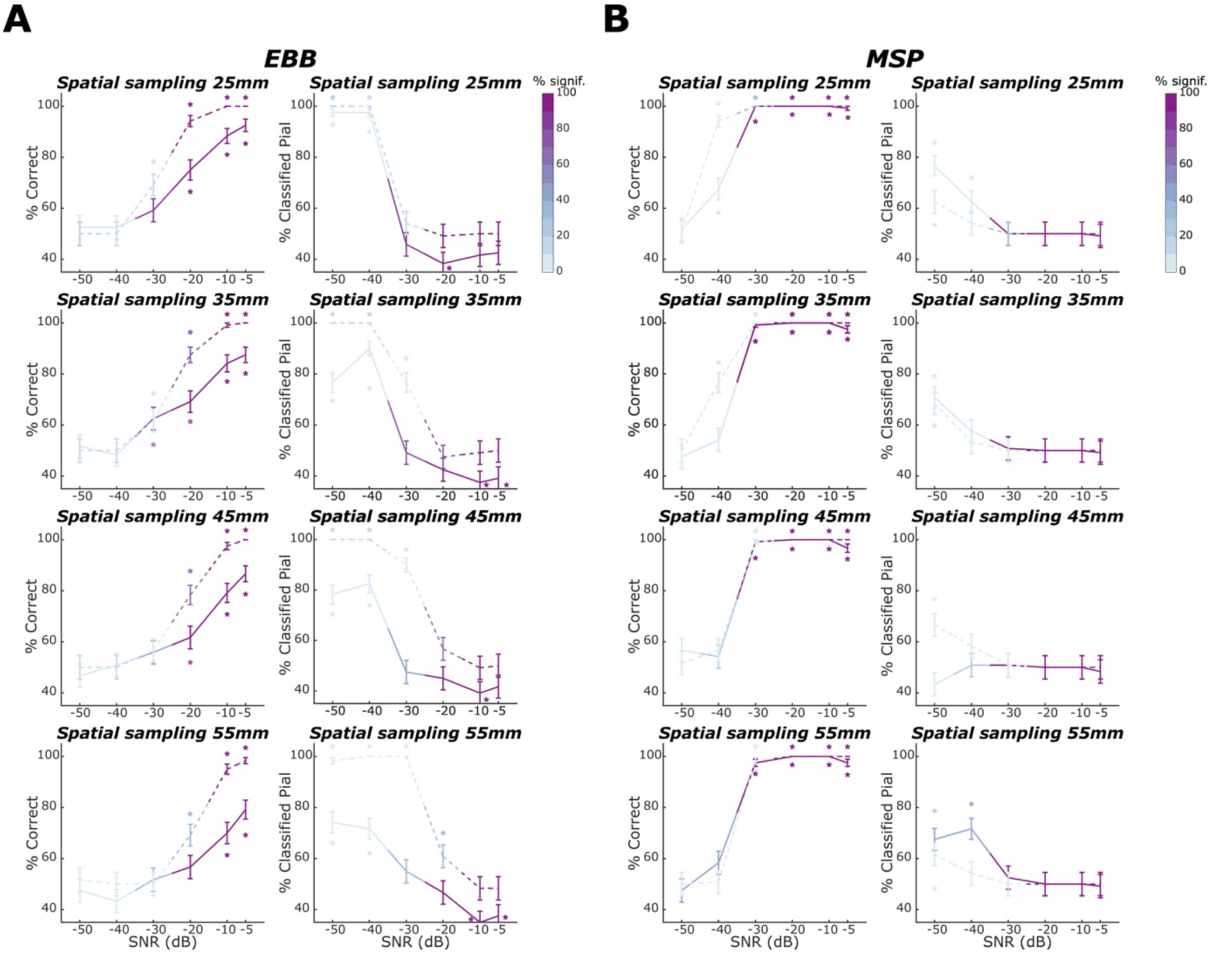

Fig. S1. Laminar classification accuracy and bias across signal-to-noise ratios across OPM-MEG sensor arrays with varying inter-sensor distances. Solid lines represent the whole-brain free energy analysis, and dashed lines represent the ROI-t-statistic analysis. The left column within each subpanel shows correct laminar inferences; the right column shows inferences favouring the pial source model. Line colour intensity indicates the percentage of simulations exceeding the significance threshold. Error bars represent standard error. Asterisks indicate significant deviations from chance levels. Spatial sampling density decreases across rows. **A** For the EBB approach, we found significant increases in classification accuracy with increase in SNR for all spatial sampling distances. **B** For the MSP approach, we observed an excellent classification performance with accuracy at ceiling and no biases for SNRs of -30 dB or higher for both, free energy and ROI-t-statistic analyses.

Laminar inference with a 3-shell BEM volume conduction model

In the simulations in the main manuscript, we used the Nolte single shell forward model due to its widespread use in MEG source inversion and its good accuracy (Stenroos et al., 2014). Here, we further investigate the potential advantage of using a high-accuracy 3-shell boundary element model (BEM) to compute the forward model. As SPM12 does not offer BEM-based forward models for MEG source reconstruction, we performed some minor adaptations to SPM's sim_eeg_inv_forward function to be

able to apply a 3-shell BEM not only to EEG but also to MEG data by using the OpenMEEG 2.4.0 package (Gramfort et al., 2010; Kybic et al., 2005). We used the default settings to calculate the volume conductor with the following conductivity values: brain: 0.33 S/m, skull: 0.0042 S/m, scalp: 0.33 S/m. Surface meshes for the inner skull boundary, the skull-scalp interface, and the scalp-air interface were generated with 2,562 vertices.

Results for an OPM sensor array with an inter-sensor distance of 55 mm and single-axis sensors are summarised in Fig. S2. Overall, classification accuracy was comparable for the 3-shell BEM and the Nolte single shell model, except for the EBB source reconstruction approach in combination with the ROI-based analysis, where the Nolte single shell model outperformed the BEM model at high SNRs (two-sided exact McNemar's tests: $p < 0.001$ at SNRs of -5 and -10 dB). While we found a strong bias towards the pial surface for the Nolte model, the bias flipped to the deep surface for the BEM forward model when using the EBB source reconstruction approach with the ROI-based analysis. Two-sided exact McNemar's tests revealed that this difference in bias towards the pial surface was significant at SNRs of -20 dB or lower (all $p < 0.0001$).

For the EBB source reconstruction approach combined with the whole-brain analysis, classification accuracy did not differ significantly, but bias was reduced for the BEM model compared to the Nolte single shell model (two-sided exact McNemar's tests: $p < 0.01$ at SNRs of -5 and -10 dB; $p < 0.05$ at an SNR of -50 dB), and no significant bias towards the pial surface was found at high SNRs for the BEM model.

For the MSP approach, no significant differences in classification accuracy were observed between the Nolte single shell and the BEM model. We found that bias was reduced for the BEM model compared to the Nolte model for the whole-brain analysis at very low SNRs (two-sided exact McNemar's tests: $p < 0.001$ at an SNR of -40 dB, and $p < 0.0001$ at an SNR of -50 dB. However, laminar inference was not feasible at these SNR levels.

The increased classification accuracy for the Nolte single shell compared to the high-accuracy 3-shell BEM model when using the EBB source reconstruction together with the ROI-based analysis may seem surprising at first glance. We assume this difference in performance may be due to the BEM forward model leading to a stronger "smearing" of the current sources on the head surface by modelling two boundaries with strong conductivity differences. This makes laminar inference more challenging than for the more focal sensor data generated with the Nolte single shell model. The degree of source spreading depends on the conductivity ratios used in the BEM forward model.

The reduced classification bias for the EBB source reconstruction approach combined with the whole-brain analysis highlights the potential for further improvement in laminar inference with a 3-shell BEM model. We note that, beyond excellent segmentation of head tissues, the accuracy of a BEM head model depends on the accurate knowledge of conductivities.

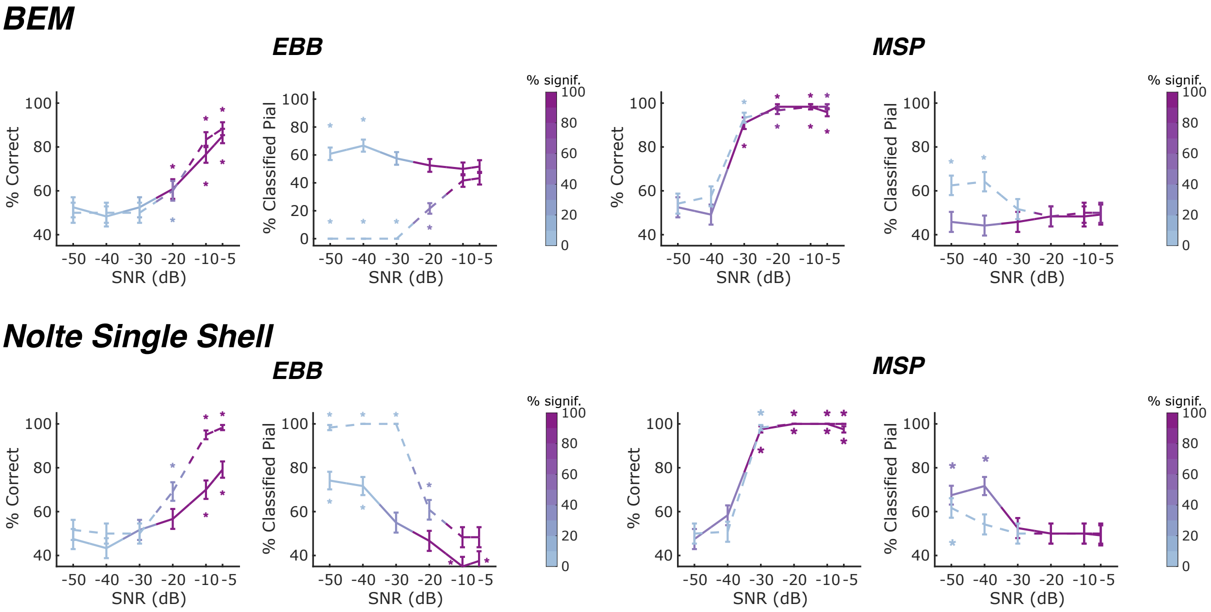

Fig. S2. Comparing laminar inference performance for the 3-shell BEM and the Nolte single shell forward models for an OPM sensor array with an inter-sensor distance of 55 mm and single-axis sensors. Solid lines represent the whole-brain free energy analysis, and dashed lines represent the ROI-t-statistic analysis. The left column shows correct laminar inferences; the right column shows inferences favouring the pial source model. Line colour intensity indicates the percentage of simulations exceeding the significance threshold. Error bars represent standard error. Asterisks indicate significant deviations from chance levels.

Laminar inference with Minimum Norm Estimates and LORETA source reconstruction approaches

We replicate previous findings that source reconstruction approaches without sparsity constraints, like Minimum Norm Estimates and LORETA, were not able to recover the laminar origin of the simulated sensor data (Fig. S3). Even under highly advantageous conditions, for a dense OPM-MEG array with an inter-sensor distance of 25 mm and simulated sensor activity at a high SNR of -5 dB, the minimum norm and LORETA source reconstruction algorithms were not able to correctly infer the laminar origin of simulated source activity using neither the whole-brain analysis (MNE, in SPM labelled IID: correct = 54.17%, p = n.s.; LORETA, in SPM labelled COH: correct = 54.17%, p = n.s.) nor the ROI analysis (IID: correct = 50.00%, p = n.s.; COH: correct = 50.00%, p = n.s.). The whole-brain analysis was biased towards the deep surface (IID: white matter = 79.17%, p <0.0001; COH: white = 80.83%, p <0.0001), and the ROI-based analysis strongly towards the superficial surface (IID: pial = 100%, p <0.0001; COH: pial = 100%, p <0.0001). The directions of these biases replicate the findings in Bonaiuto et al., 2018.

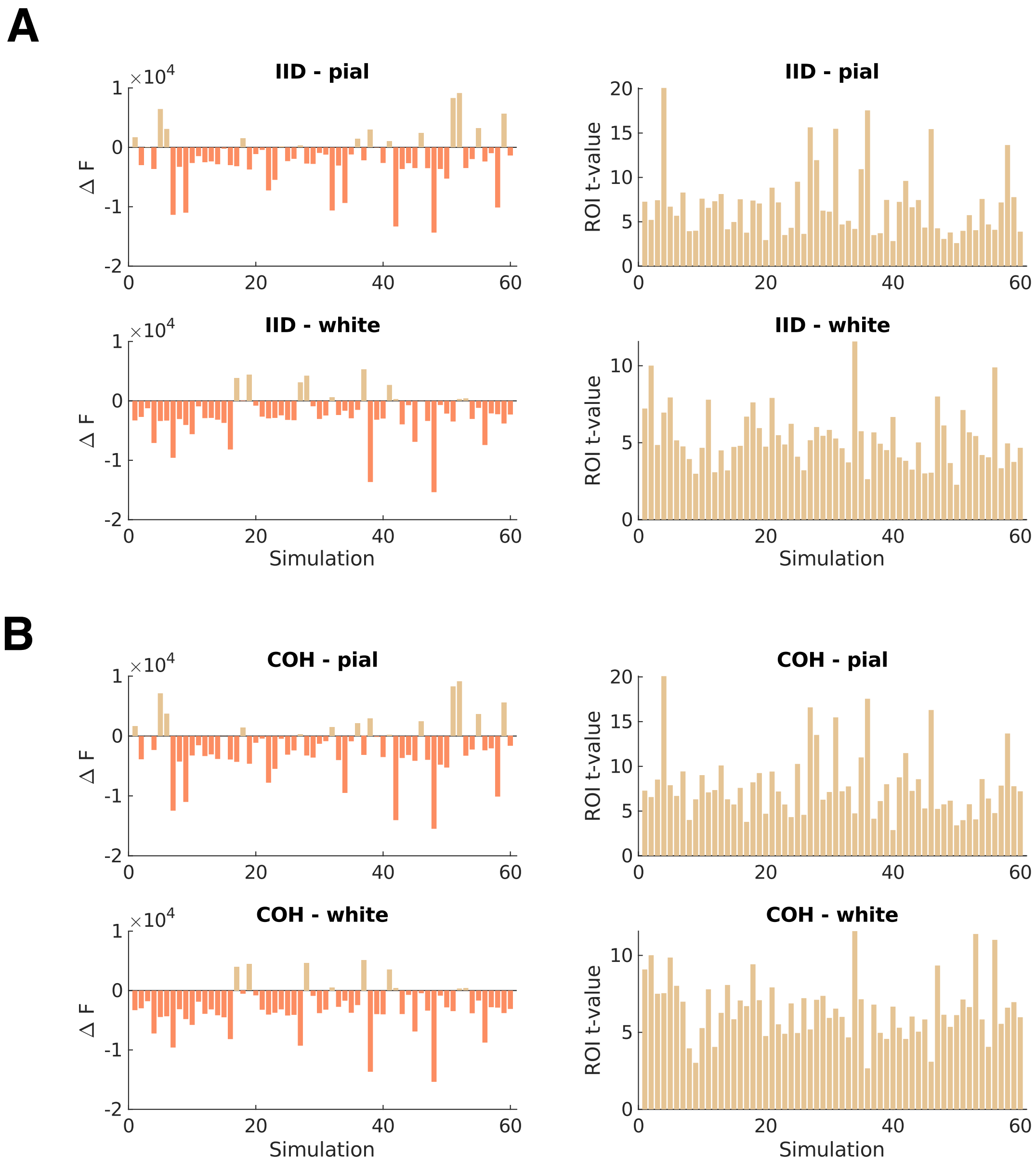

Fig. S3. The source reconstruction approaches without sparsity constraints, i.e., MNE (IID) and Loreta (COH), were not able to perform laminar source discrimination. We used a dense OPM-MEG array with an inter-sensor distance of 25 mm and simulated sensor activity at a relatively high SNR of -5 dB. Left column: The difference in free energy between the pial and white matter generative models in each simulation (SNR = -5 dB). Right column: T-statistics from the ROI analysis comparing pial and white matter ROIs for each simulation (SNR = -5 dB). Each panel shows simulations with pial surface sources on the top row, and simulations with white matter surface sources on the bottom row. The minimum norm estimate (**A**; IID) and LORETA (**B**; COH) source reconstruction algorithms were not able to correctly classify simulated source activity as originating from either the deep or superficial surface for the whole-brain or the ROI analysis. The whole-brain analysis was biased towards the deep and the ROI analysis strongly towards the superficial surface.

Increasing the number of measurement axes for OPM-MEG arrays with denser spatial sampling

While we observed significant increases in classification accuracy with increasing number of measurement axes for an OPM-MEG sensor array with an inter-sensor distance of 55 mm, we acknowledge that such an effect may have been particularly strong at low sensor sampling densities

where the advantage of a more homogenous spatial coverage afforded by sensors with multiple axes is expected be more pronounced. We thus re-analysed the impact of the number of measurement axes on our ability to perform laminar inference with a denser OPM-MEG array with a 35 mm inter-sensor distance and report results for 69-, 134- and 207-channel configurations for single-axis, dual-axis and triaxial sensor arrays, respectively. Note that the channel count for the dual-axis sensor array deviates from being twice the sensor count of the single-axis array due to a random factor in the point packing algorithm. The point packing algorithm was re-initialized for each array configuration, leading to the observed deviations in channel counts.

For the EBB approach combined with the free energy analysis, classification performance increased with the number of measurement axes at SNRs of -20 dB or higher. However, these increases were not significant, and only trends of increasing classification accuracy were observed at -5 dB (beta = -0.603, p = 0.078) and -20 dB (beta = -0.389, p = 0.080). We again found a bias towards the deep surface, as described previously for the single-axis configuration. This bias did not systematically increase or decrease with an increase in the number of measurement axes. We found no advantage of increasing the number of measurement axes at -30 dB SNR and no significant changes in classification bias across measurement axes. At a very low SNR of -40 dB, laminar inference was not feasible, regardless of the number of measurement axes.

For the ROI-based analysis, classification performed at ceiling for SNRs of -5 and -10 dB and increased significantly with the number of measurement axes at -20 dB (beta = -1.086, $p < 0.05$). At an SNR of -30 dB, we observed a trend of increased classification accuracy with an increase in the number of axes (beta = -0.409, p = 0.051), while the bias towards the pial surface reduced with an increase in the number of measurement axes (beta = 0.641, $p < 0.01$). However, laminar inference was not statistically significant at the single-simulation level. At a very low SNR of -40 dB, classification accuracy was at chance level with a bias towards the pial surface, which increased with the number of measurement axes (beta = -1.462, $p < 0.01$). Note that laminar inferences were not significant at the single-source level.

For the MSP approach, both free energy and ROI-based analysis performed at ceiling for SNRs of -30 dB or higher, irrespective of the number of measurement axes. In contrast, at an SNR of -40 dB, classification accuracy increased strongly with the number of measurement axes (whole-brain: beta = -0.644, $p < 0.01$, ROI: beta = -2.186, $p < 0.001$); however, these laminar inferences did not exceed the significance threshold at the single-source level.

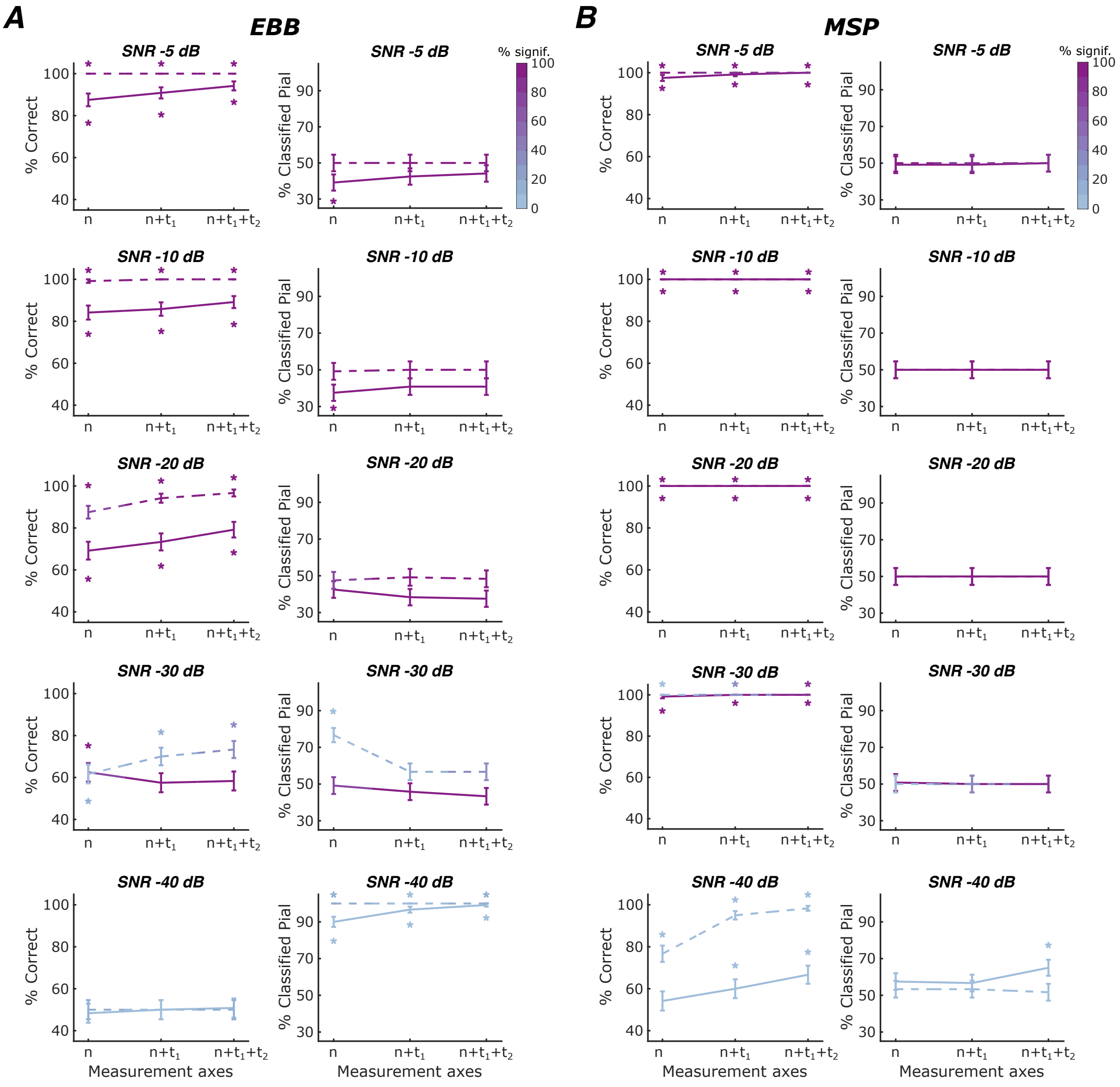

Fig. S4. Laminar classification accuracy and bias across number of measurement axes for an OPM-MEG array with a sensor spacing of 35 mm. OPM sensor arrays were modelled with one to three measurement axes: a single radial axis oriented normally to the scalp surface (n), a radial axis and a tangential axis ($n+t_1$), a radial axis and two orthogonal tangential axes ($n+t_1+t_2$). Solid lines represent the whole-brain free energy analysis, and dashed lines represent the ROI-t-statistic analysis. The left column within each subpanel shows correct laminar inferences; the right column shows inferences favouring the pial source model. Line colour intensity indicates the percentage of simulations exceeding the significance threshold. Error bars represent standard error. Asterisks indicate significant deviations from chance levels. SNR decreases across rows. **A** For the EBB approach, we found increases in classification accuracy with increasing numbers of measurement axes at SNRs of -20 dB or higher. However, these changes did not reach statistical significance. **B** For the MSP approach, classification performance was at ceiling and did not vary significantly with the number of measurement axes at all SNRs at which laminar inference was possible.

Interfering brain noise sources: 50 internal noise sources at a relative source strength of 0.1

We further investigated the impact of internal noise sources on laminar inference performance by running an additional set of simulations with 50 brain noise sources. As for the simulations with five brain noise sources presented in the main manuscript, we simulated the sensor activity for a dense OPM-MEG array with an inter-sensor distance of 25 mm and single-axis sensors. The sensor activity from a laminar cortical source of interest was again modelled at an SNR of -5 dB. We then added 50 concurrent, weaker noise sources on the mid-cortical surface at a lower relative source strength of 0.1. The average single-trial SNR was -28.28 dB (std: ± 6.03 dB) and the average SNR of the trial averaged data was -27.58 dB (std: ± 6.39 dB). The results are summarized in Fig. S5.

For the EBB source reconstruction approach combined with the whole-brain analysis, we were not able to infer the laminar origin of the simulated sources (correct = 50.83%, p = n.s.) and observed a strong bias towards the deep surface (white matter = 99.17%, $p < 0.00001$). For the ROI analysis, we observed significant classification accuracy (EBB: correct = 70.83%, $p < 0.00001$), which, however, was biased towards the superficial surface (pial = 67.50%, $p < 0.001$). For the MSP source reconstruction approach, the whole-brain analysis showed low classification accuracy, though still above chance level (correct = 63.33%, $p < 0.01$), and laminar inference was biased towards the deep surface (white matter = 86.67%, $p < 0.00001$). For the ROI analysis, the MSP approach yielded a higher classification accuracy (correct = 75.00%, $p < 0.00001$) and showed no classification bias (pial = 51.67%, n.s.).

In summary, we were only able to perform laminar inference without significant biases when using the MSP source reconstruction approach combined with the ROI analysis.

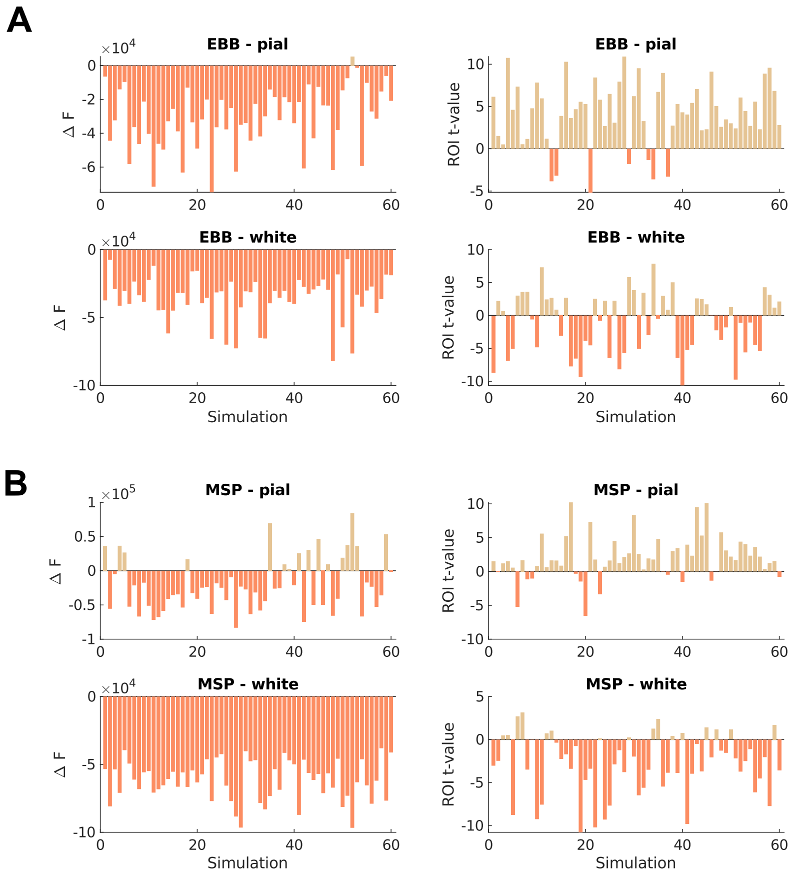

Fig. S5. Laminar source discrimination in the presence of 50 interfering brain noise sources with a relative source strength of 0.1. We used a dense OPM-MEG array with an inter-sensor distance of 25 mm and simulated sensor activity at a relatively high SNR of -5 dB. Results are shown for the **A** EBB and **B** MSP source reconstruction approaches. Left column: the difference in free energy between the pial and white matter generative models in each simulation. Right column: t-values for the difference between pial and white matter ROI values for each simulation. Each panel shows simulations with pial surface sources on the top row, and simulations with white matter surface sources on the bottom row.

Supplementary references

Bonaiuto, J. J., Rossiter, H. E., Meyer, S. S., Adams, N., Little, S., Callaghan, M. F., Dick, F., Bestmann, S., & Barnes, G. R. (2018). Non-invasive laminar inference with MEG: Comparison of methods and source inversion algorithms. *NeuroImage*, *167*, 372–383. https://doi.org/10.1016/j.neuroimage.2017.11.068

Gramfort, A., Papadopoulo, T., Olivi, E., & Clerc, M. (2010). OpenMEEG: Opensource software for quasistatic bioelectromagnetics. *BioMedical Engineering OnLine*, *9*(1), 45. https://doi.org/10.1186/1475-925X-9-45

Kybic, J., Clerc, M., Abboud, T., Faugeras, O., Keriven, R., & Papadopoulo, T. (2005). A common formalism for the Integral formulations of the forward EEG problem. *IEEE Transactions on Medical Imaging*, *24*(1), 12–28. IEEE Transactions on Medical Imaging. https://doi.org/10.1109/TMI.2004.837363

Stenroos, M., Hunold, A., & Haueisen, J. (2014). Comparison of three-shell and simplified volume conductor models in magnetoencephalography. *NeuroImage*, *94*, 337–348. https://doi.org/10.1016/j.neuroimage.2014.01.006
